# Neuronal differentiation and activity drive nucleocytoplasmic shuttling of the intellectual disability kinase TLK2

**DOI:** 10.3389/fncel.2026.1699735

**Published:** 2026-04-07

**Authors:** Lubna Nuhu-Soso, Heidi Denton, Darren L. Goffin, Ines Hahn, Gareth J. O. Evans

**Affiliations:** York Biomedical Research Institute, Department of Biology, University of York, York, United Kingdom

**Keywords:** kinase, neurodevelopmental disorder, neuronal differentiation, nuclear localisation signal, splice variant

## Abstract

**Introduction:**

Autosomal dominant intellectual developmental disorder 57 (MRD57) is a rare neurodevelopmental disorder characterised by delayed language and psychomotor development, intellectual disability, hypotonia, gastrointestinal issues and facial dysmorphia. It is linked to genetic mutations in the serine/threonine kinase TLK2, which generally cause haploinsufficiency. TLK2 is an established cell cycle regulator that has been extensively studied in mitotic cells. It is upregulated in cancers, driving tumour growth, however, the role of TLK2 in postmitotic neurons is not understood. We therefore aimed to determine where TLK2 is expressed in the brain and its subcellular localisation during neuronal differentiation.

**Methods:**

We analysed TLK2 transcript or protein expression and localisation in public RNAseq datasets, mouse brain sections, and a rat neuroblastoma cell line model of neuronal differentiation.

**Results:**

Human and mouse brain transcriptomic data revealed splice variant diversity in the N-terminus of TLK2, which contains its nuclear localisation sequence (NLS). Using splice-specific in situ hybridisation probes, we observed expression of TLK2 transcripts that contain and lack the NLS in the mouse hippocampus and cerebellum. Surprisingly, TLK2 protein was predominantly cytoplasmic in the adult mouse brain. Similarly, in rat neuroblastoma cells, we found that neuronal differentiation enhances a cytoplasmic pool of TLK2 by two mechanisms: nuclear export of full length TLK2 and increased expression of TLK2 splice variants lacking the NLS. Finally, acute stimuli that mimic synaptic activity were sufficient to elicit nuclear export of TLK2.

**Discussion:**

Our data highlight a previously unrecognised role of cytoplasmic TLK2 in neurons and future studies should determine how the loss of TLK2 activity in MRD57 impacts cytoplasmic TLK2 substrates in the developing and mature brain.

## Introduction

Tousled-like kinase 1 and 2 are the mammalian homologues of the *Arabidopsis* mutant, *tousled*, which was discovered in a developmental screen (Roe et al., 1993). Indeed, both human TLK1 and TLK2 have been linked with rare developmental disorders (Segura-Bayona and Stracker, 2019; Villamor-Payà et al., 2024). To date, there have been close to 50 documented patients worldwide with TLK2 mutations, classified as autosomal dominant intellectual developmental disorder 57 (MRD57; Lelieveld et al., 2016; Reijnders et al., 2018; Töpf et al., 2020; Pavinato et al., 2022; Woods et al., 2022), with a predicted incidence of approximately 3 in 100,000 individuals (López-Rivera et al., 2020). The clinical phenotypes associated with MRD57, like other neurodevelopmental disorders, are heterogeneous, including intellectual disability, delayed psychomotor development in infancy or early childhood, language delay, hypotonia, feeding problems, gastrointestinal issues, dysmorphic facial features, microcephaly, behaviour problems, and global developmental delays (Reijnders et al., 2018; Töpf et al., 2020; Pavinato et al., 2022; Woods et al., 2022). The majority of TLK2 mutations in MRD57 are *de novo* mutations, but seven are inherited within four different families (Reijnders et al., 2018; Töpf et al., 2020; Pavinato et al., 2022). Like the *de novo* cases, inherited TLK2 mutations are heterozygous dominant except one report of a homozygous recessive inheritance (Reijnders et al., 2018; Töpf et al., 2020; Pavinato et al., 2022). MRD57 patients have TLK2 mutations throughout the protein and all are predicted to result in a reduction of TLK2 expression or its catalytic activity (Mortuza et al., 2018; Reijnders et al., 2018).

Functionally, TLK2 is an established regulator of DNA replication, cell cycle checkpoint recovery and chromatin remodelling (Bruinsma et al., 2016; Kim et al., 2016a; Segura-Bayona et al., 2017; Silljé et al., 1999). TLK2 facilitates cell cycle progression mainly by enhancing histone supply via phosphorylation of its best characterised substrate, ASF1 (Klimovskaia et al., 2014). In-line with other cell cycle kinases, TLK activity and gene copy number are increased in cancer, including breast, cervix, lung, liver, colon and kidney (Bhoir and De Benedetti, 2023; Kim et al., 2016b; Lee et al., 2018). The chromosomal locus of TLK2, 17q23, contains other oncogenes, and is frequently amplified in more than 40% of breast cancer tumours (Kelemen et al., 2009; Kim et al., 2016b). Moreover, single nucleotide polymorphisms (SNPs) in TLK2 have been identified as risk factors for breast cancer (Kelemen et al., 2009). Indeed, inhibition of TLK2 has been identified as a therapeutic strategy and small molecule inhibitors are in development (Kim et al., 2016b; Lee et al., 2018).

In contrast to its role in the cell cycle and cancer, the neuronal expression and cell biology of TLK2 is poorly understood. Here we have characterised its neuronal transcript expression, revealing a conserved splice diversity in rodents and humans. TLK2 alternative splicing occurs in exons encoding the N-terminus, resulting in ‘long’ and ‘short’ isoforms, with the latter lacking the nuclear localisation sequence (NLS). Expression of both long and short TLK2 transcripts is prevalent in neurons of the mouse hippocampus and cerebellum. Surprisingly, TLK2 protein staining was mainly detected in the cytoplasm of adult mouse cerebellar and hippocampal neurons. Using a cell model of neuronal differentiation, we observed the upregulation of short TLK2 expression during neuronal differentiation, with a concomitant shuttling of long TLK from the nucleus to the cytoplasm. Pharmacological treatments mimicking neuronal activity also drive nucleocytoplasmic shuttling of TLK2. These data highlight the likely importance of cytoplasmic TLK2 in terminally differentiated neurons, which have exited the cell cycle. There is hence a need to determine the neuronal substrates of TLK2 to shed light on the neuronal phenotypes of MRD57.

## Materials and methods

### Materials

Rat B104 and human Flp-In T-REx SK-N-SH neuroblastoma cell lines were kind gifts from Dr. Martin Rumsby, University of York and Dr. Han-Jou Chen, University of York, respectively. The Flp recombinase expression vector, pOG44 was a kind gift from Dr. Paul Pryor, University of York.

### Bioinformatics

TLK2 transcript IDs and exon structures were derived from their Ensembl gene annotations (version 113) using human (GRCh38.p14) or mouse (GRCm39) genome builds (Harrison et al., 2024). The human TLK2 RNAseq data used for the transcriptomic analyses described in this manuscript were obtained from the GTEx Portal, accession number phs000424.v8.p2. Mouse brain TLK2 RNAseq data was obtained from the Human Protein Atlas (proteinatlas.org; Sjöstedt et al., 2020). TLK2 transcript expression values in transcripts per million (TPM) were converted to percentages by tissue or brain region.

### RNA extraction, cDNA synthesis and RT PCR

All work involving mice was approved by the University of York Animal Welfare and Ethical Review Body and performed under UK Home Office legislation (project licence P38B2013E).

Adult wild-type C57BL/6 mouse forebrain and cerebellum were dissected and homogenised in RIPA buffer [50 mM Tris, pH 8.0, 150 mM NaCl, 1% (v/v) Triton-X-100, 0.5% (w/v) sodium deoxycholate, 0.1% (w/v) SDS, 1 mM EDTA supplemented with 1 mM Na_3_VO_4_, 0.1% (v/v) β-mercaptoethanol, 1 mM PMSF and 1:200 protease inhibitor cocktail (Sigma)] using 5 mL buffer/g of tissue. The homogenate was incubated at 4 °C with agitation for 1 h and then centrifuged at 20,000 *g* for 30 min at 4 °C. RNA was extracted from the supernatant.

B104 cells undergoing neuronal differentiation were plated at 10^4^ cells/ cm^2^ onto collagen coated 6-well tissue culture dishes. RNA was extracted from the differentiating cells at 2 days intervals. For both mouse brain and B104, RNA was extracted using a NucleoSpin RNA isolation kit (Macherey-Nagel) according to the manufacturer’s instructions.

Complementary DNA was synthesised from 1 μg of RNA using a SuperScript IV First-Strand cDNA Synthesis Kit (Life Technologies) according to the manufacturer’s instructions in the presence of both oligo d(T)_18_ and random hexamer primers. A no RT cDNA sample was performed for each time point and cDNA was stored at −20 °C. PCR reactions were performed in 25 μL reactions containing 1 μL of cDNA, 1X GoTaq flexi buffer, 0.2 mM each of dNTP mix, 0.4 μM of each primer pair (Table 1) and 1 U Taq polymerase. Cycling conditions consisted of an initial denaturing step at 98 °C for 30s followed by 25–30 cycles of 95 °C for 10s, 55 °C for 30s, 72 °C for 1 min/kb and a final extension at 68 °C for 10 min. 5–10 μL of the PCR products were separated on 1.5%–2% (w/v) agarose gels to confirm amplification.

**Table 1:** Primers and probes used in this study.

Name	Sequence (5′-3′)
PCR primers	
mTLK2-exon1 sense	GACCCACGAAGGCAGGAATTA
mTLK2-exon5/6 anti	CTCGACGCGGTAAGGGATTG
mTLK2-exon4/6 anti	CTGCTCGACGCTCAAAGTAA
mTLK2-exon3/6 anti	GCTCGACGCTTGGCTAGT
mTLK2-exon6 anti	GCTGCACTGCCATCTAAACC
mGAPDH sense	CATCACTGCCACCCAGAAGACTG
mGAPDH anti	ATGCCAGTGAGCTTCCCGTTCAG
hTLK2-exon1 sense	ATTGCATAGCCTGGACCCAC
hTLK2-exon9 anti	TCGTTCCTTGTATTTCTCTAGCA
hMAPT sense	ACACCATGCACCAAGACCAA
hMAPT anti	TCCTTCTGGGATCTCCGTGT
hGAPDH sense	GTCTCCTCTGACTTCAACAGCG
hGAPDH anti	ACCACCCTGTTGCTGTAGCC
ISH probes	
mTLK2-204	GGAACCAGCCCTGGCAGAAGTGTTCCACCAGTTGCACGATCCTCACCGCAACATTCCTTATCCAATCCCTTACCGCGTCGAGCAGAACAGCCTCTGTATGGTTTAGATGGCAGTGCAGCAAAGGAGGCCTCAGAAGAGCAGTCTGCTCTG
mTLK2-201	AGGAAAGCCGAGCCATATGACACTAGCCAAGGGAAAGGCACTCCTAGGGGACATAAAATTAGTGATTACTTTGAGCGTCGAGCAGAACAGCCTCTGTATGGTTTAGATGGCAGTGCAGCAAAGGAGGCCTCAGAAGAGCAGTCTGCTCTG
mTLK2-207	TAGAGACTCCTGAGAAAAAGCAGAATGACCAGCGAAATCGGAAAAGGAAAGCCGAGCCATATGACACTAGCCAAGCGTCGAGCAGAACAGCCTCTGTATGGTTTAGATGGCAGTGCAGCAAAGGAGGCCTCAGAAGAGCAGTCTGCTCTG
mTLK2-novel	CAACCAGAGTCTGTGCAGCGTGGGGTCGTTGAGTGATAAAGAAGTAGAGACTCCTGAGAAAAAGCAGAATGACCGCGTCGAGCAGAACAGCCTCTGTATGGTTTAGATGGCAGTGCAGCAAAGGAGGCCTCAGAAGAGCAGTCTGCTCTG
mCalb1	GATGCTTTGCTGAAAGATCTGTGTGAGAAGAACAAACAGGAATTGGATATTAACAATATTACTACATACAAGAAGAACATAATGGCCTTGTCGGATGGAGGGAAGCTGTACCGAACAGACCTTGCTCTTATTCTTTCTGCTGGAGACAAC

### Preparation of mouse brain sections

Adult wild-type C57BL/6 mice were anaesthetised with isoflurane inhalation followed by an intraperitoneal injection of 100 mg/kg ketamine and 12.5 mg/kg xylazine and then transcardially perfused with 4% paraformaldehyde (PFA) in 0.1 M sodium-potassium PBS. The brains were dissected and post-fixed in 4% PFA overnight at 4 °C. Following a further 24 h incubation in 30% sucrose, brains were embedded in OCT, frozen at −80 °C, and 25 μm coronal or sagittal sections were cut using a Leica CM1950 cryostat.

### *In situ* hybridisation

Probes for *in situ* hybridisation were transcribed using 10X digoxigenin (DIG) RNA labelling mix (Roche) and T7 polymerase. For *in vitro* transcription, template DNA was synthesised as gBlocks™ (IDT) and PCR amplified to introduce the T7 promoter sequence (Table 1). *In situ* hybridisation was performed on adult mouse sagittal or coronal brain sections according to (Fisher et al., 2002) with adaptations for sections mounted on slides. Briefly, the sections were first incubated in 4% PFA for 10 min at room temperature (RT) followed by two 5 min PBST washes. Sections were then treated with 4 μg/mL proteinase K (Roche) in PBST for 10 min and then washed twice in PBST for 5 min. A second fix in 4% (w/v) PFA was performed for 10 min, followed by two 5 min PBST washes. Next, the sections were acetylated using 0.5% (v/v) acetic anhydride in 0.1 M triethanolamine, pH 7.8 for 10 min followed by two 5 min PBST washes and finally a 15 min wash in 5X saline-sodium citrate buffer (SSC; Sigma). Pre-hybridisation was carried out by incubating the sections in hybridisation buffer (50% (v/v) formamide (Ambion, AM9342), 5X SSC, 100 μg/mL heparin, 1X Denhardt’s, 0.1% (v/v) Tween-20, 0.1% (w/v) CHAPS, 10 mM EDTA, 1 mg/mL total yeast RNA) containing no probe at 65 °C for 2–5 h in a humidified box. Hybridisation followed by incubating the sections with 300 ng/mL of probe solution (DIG prep in hybridisation buffer) overnight at 65 °C in a humidified box.

Post-hybridisation, the sections were subjected to several hot wash steps. First a 10 min wash in hybridisation buffer containing no probe at 65 °C, then a 1 h wash in 2X SSC followed by a 1 h wash in 0.2X SSC, both at 65 °C. The sections were then washed twice for 5 min in MABT (100 mM maleic acid, 150 mM NaCl, 0.1% (v/v) Tween-20, pH 7.8) at RT and then blocked in blocking solution [2% (w/v) Boehringer Mannheim Blocking Reagent (Roche, 11096176001), 20% (v/v) heat-treated lamb serum (Sigma), 1X MAB] for 2 h at RT in a humidified box. Next, the sections were incubated with anti-DIG-AP (Table 2) in blocking solution overnight at 4 °C in a humidified box. Post-antibody washes were carried out at RT by washing the sections three times for 1 h in MABT and once in alkaline phosphatase (AP) buffer (100 mM Tris, pH 9.5, 50 mM MgCl_2_, 100 mM NaCl) for 10 min. The colour reaction was initiated by adding a 1:3 dilution of BM purple (Roche, 11442074001) in AP buffer to the sections and incubation at RT in a humidified box for 1–5 days. The reaction was stopped by washing the sections twice for 10 min in PBST, fixing in 4% (w/v) PFA for 1 h or overnight at 4 °C and a 15 min wash in PBST. The sections were air dried, mounted in aqueous mounting media (Sigma, 324590) and stored at 4 °C. Images were acquired on a Leica DM2500 microscope with a 10X objective (sagittal) and a Zeiss Stemi 508 stereo microscope (coronal).

**Table 2:** Antibodies used in this study.

Name	Dilution (application)	Species	Catalogue no.	Supplier
Primary antibodies				
Anti-TLK2	1:1,000 (WB) 1:500 (IF)	Rabbit	A301-257A	Bethyl
Anti-TLK2	1:200 (IHC)	Rabbit	13979-1-AP	Proteintech
Anti-TORC1/CRTC1	1:1,000 (IF)	Rabbit	2587	Cell Signaling Technology
Anti-β-III-tubulin	1:1,000 (WB) 1:500 (IF)	Mouse	801202	Biolegend
Anti-β-actin	1:50,000–1:100,000 (WB)	Mouse	60008-1-Ig	Proteintech
Anti-Histone H3	1:5,000 (WB)	Mouse	819411	Biolegend
Anti-GAPDH	1:5,000 (WB)	Mouse	MAB374	Sigma
Anti-FLAG	1:1,000 (IF/WB)	Mouse	F1804	Sigma
Anti-Digoxigenin	1:2,000 (ISH)	Sheep	11093274910	Roche
Secondary antibodies				
Anti-mouse HRP	1:5,000 (WB)	Goat	A4416	Sigma
Anti-rabbit HRP	1:5,000 (WB)	Goat	A6154	Sigma
Anti-mouse Alexa 594	1:500 (IF)	Goat	A11020	Invitrogen
Anti-rabbit Alexa 488	1:500 (IF)	Goat	A11008	Invitrogen
Anti-rabbit Alexa 594	1:500 (IHC)	Goat	A11012	Invitrogen

### Immunohistochemistry

Before immunostaining, heat-induced epitope retrieval was performed on adult mouse brain sagittal sections. Sections were incubated with 10 mM sodium citrate buffer, pH 6.0 containing 0.05% (v/v) Tween-20 for 15 min at 90 °C and then allowed to cool down for approximately 35 min. The sections were washed twice in PBS and then permeabilised by incubating in PBS containing 0.2% (v/v) TritonX-100 for 10 min and then PBS containing 10% (v/v) methanol for 5 min. Sections were then blocked in PBS containing 3% (v/v) normal goat serum (NGS) for 1 h at RT. Primary antibody was applied overnight at 4 °C in PBS containing 1% (v/v) NGS, 0.1% (w/v) sodium azide and 0.2% (v/v) TritonX-100. Sections were washed three times for 5 min in PBS and then incubated with fluorescent secondary antibodies for 2 h at RT. Next, sections were washed twice for 5 min in PBS and then incubated in PBS containing 2 μg/mL Hoechst-33342 for 5 min. Finally, sections were washed once in PBS for 5 min and then twice for 5 min in phosphate buffer (80.5 mM K_2_HPO_4_, 19.5 mM NaH_2_PO_4_) and mounted in ProLong Gold. Images were acquired in the Bioscience Technology Facility (University of York) on a Zeiss Observer 7 epi-fluorescence microscope.

### Culture, neuronal differentiation and pharmacological treatment of B104 cells

B104 cells were maintained at 37 °C, 5% CO_2_ in a humidified atmosphere in Dulbecco’s Modified Eagle Medium (DMEM) with high glucose, pyruvate and L-glutamine supplemented with 10% foetal bovine serum (FBS) and 1% penicillin/streptomycin.

Neuronal differentiation of B104 cells was performed according to a previously described method (Encinas et al., 2000). Briefly, tissue culture dishes or coverslips were coated in 0.05 mg/mL collagen in 60% (v/v) ethanol. Cells were plated at 10^4^ cells/cm^2^ (for protein analysis) or 5 × 10^3^ cells/well (for microscopy) in DMEM supplemented with 15% (v/v) heat inactivated FBS and 1% (v/v) penicillin/streptomycin. Twenty-four hours after plating, the media was changed and supplemented with 10 μM retinoic acid (RA; Sigma, R2625). Six days after plating, the cells were washed three times in serum-free DMEM and then incubated with serum-free DMEM containing 50 ng/mL brain-derived neurotrophic factor (BDNF; Qkine) and 1% (v/v) penicillin/streptomycin and cultured for a further 7 days. Throughout the differentiation process, the media was changed every 2 days and supplemented with the appropriate differentiation agent.

For analysing protein expression in differentiating cells, lysates were collected at 2 day intervals. Cells were washed twice in sterile ice-cold PBS and scraped in 100 μL of lysis buffer (20 mM HEPES, pH 7.4, 10 mM KCl, 2 mM MgCl_2_, 1 mM EDTA and 1 mM EGTA, 1 mM DTT and protease inhibitor cocktail). Lysates were incubated on ice for 15 min, passed through a 25G needle 10 times and protein concentration was determined by Bradford assay. The lysates were diluted in Laemmli sample buffer, boiled for 10 min at 90 °C and 10 μg of protein was separated by SDS-PAGE and analysed by Western blotting.

Pharmacological stimulation of undifferentiated B104 cells was performed on cells plated 24 h prior to treatment on 13 mm coverslips in a 24-well plate at 5 × 10^4^ cells/well. Cells were washed twice in PBS and then incubated for 1 h at 37 °C, 5% CO_2_ in either control buffer [170 mM NaCl, 3.5 mM KCl, 0.4 mM KH_2_PO4, 10 mM HEPES, 5 mM NaHCO_3_, 5 mM glucose, 1.2 mM Na_2_SO_4_, 1.2 mM MgCl_2_, 1.3 mM CaCl_2_, pH 7.4, 0.2% (v/v) DMSO] or KCl/FSK buffer [control buffer with 73.5 mM NaCl, 100 mM KCl, 10 μM forskolin, 100 μM IBMX, 0.2% (v/v) DMSO]. Cells were then fixed, and immunocytochemistry was performed as described below using anti-TLK2 or anti-TORC1/CRTC1.

### Inducible stable TLK2 cell lines

Tetracycline-inducible SK-N-SH cell lines expressing either full length TLK2-203 or short N-terminally truncated TLK2-213 were generated using the Flp-In™ T-REx™ System (Invitrogen). pcDNA5/FRT/TO-FLAG-TLK2 was purchased from the MRC PPU Reagents and Services facility (College of Life Sciences, University of Dundee). TLK2-213 was made by subcloning its ORF from full length TLK2 and ligating it back into the same backbone via 5′ and 3′ NotI restriction sites with the N-terminal FLAG tag intact. Cells were co-transfected with the FRT backbone containing the gene of interest (TLK2-203 or TLK2-213) and a Flp-recombinase expression vector (pOG44). Positive clones were selected by treating with 0.5 mg/mL hygromycin B and assaying for protein expression after the addition of 1 μg/mL doxycycline.

### Subcellular fractionation

Flp-In SK-N-SH cells were fractionated according to the Abcam subcellular fractionation protocol. TLK2 expression was induced using 1 μg/mL doxycycline and left in culture for 48 h prior to fractionation. Briefly, cells were scraped in 500 μL of lysis buffer (described above) and incubated on ice for 15 min. The scraped cells were passed through a 25G needle 10 times, incubated on ice for 20 min and then centrifuged at 720 *g* for 5 min at 4 °C. The post-nuclear supernatant was transferred to a fresh tube. The nuclear pellet was washed by first resuspending in lysis buffer, passing the sample through a 25G needle 10 times and centrifuging at 720 *g* for 10 min at 4 °C. The washed pellet was resuspended in TBS containing 0.1% (w/v) SDS, and sonicated on ice for 3 s. The protein concentration of each fraction was determined by Bradford assay, and 20 μg of each fraction was separated by SDS-PAGE and analysed by Western blotting.

### Western blotting

Proteins separated by SDS-PAGE (7.5%–12%) gels were transferred onto PVDF membranes (Millipore). Membranes were incubated with primary antibodies (Table 2) overnight at 4 °C with agitation. Membranes were washed and incubated with appropriate HRP-conjugated secondary antibodies and immunoreactivity was visualised by incubation with enhanced chemiluminescence reagent and imaged on an iBright FL1000 scanner (Invitrogen).

### Immunocytochemistry and fluorescence microscopy

Cells were washed 3× in PBS and then fixed in 4% (w/v) paraformaldehyde (PFA) in PBS for 20 min at RT. Next cells were washed 3× in PBS then permeabilised and blocked in PBS containing 0.1% (v/v) TritonX-100 and 1% (w/v) bovine serum albumin (BSA). Primary antibodies were diluted in PBS containing 1% (w/v) BSA at appropriate dilutions (Table 2) and incubated on cells overnight at 4 °C. Cells were washed 3x in PBS then incubated with secondary antibodies in PBS containing 1% (w/v) BSA for 1 h in the dark at RT. Finally, cells were washed 3× in PBS, 1× in dH_2_O, air dried and mounted in Mowial containing 1 μg/mL 4′,6-diamidino-2-phenylindole (DAPI). Images were acquired in the Bioscience Technology Facility (University of York) on a Zeiss LSM 880 confocal microscope on a 40×/1.4 Oil DIC III objective.

### Data analysis

Quantification of hippocampal *in situ* hybridization images was performed on 16 bit grayscale images in FIJI (Schindelin et al., 2012). The oval selection tool was used to draw regions of interest (ROI) in different locations within the hippocampal formation (CA1, CA3, DGs, and DGi). The intensity was then measured and normalised to DGs for each section and probe. Three to four hippocampi were quantified for each probe, and the values were plotted as a heatmap in Morpheus.^1^

The quantification of cytoplasmic/nuclear TLK2 ratios of immunofluorescent images was conducted by measuring the TLK2 intensity in the nucleus and cytoplasm of the cells in FIJI using the ROI 1-click plug-in (Thomas and Gehrig, 2020). Three hundred cells for each condition were analysed from three technical replicates (100 cells/replicate). Western blots were quantified by densitometry analysis in FIJI. Statistical significance was assessed by *t*-test or non-parametric ANOVA with Dunn’s *post-hoc* test. Statistical analyses and graph plotting was performed in RStudio Team (2020).

## Results

### TLK2 is alternatively spliced in human and mouse brain

**Figure 1:**
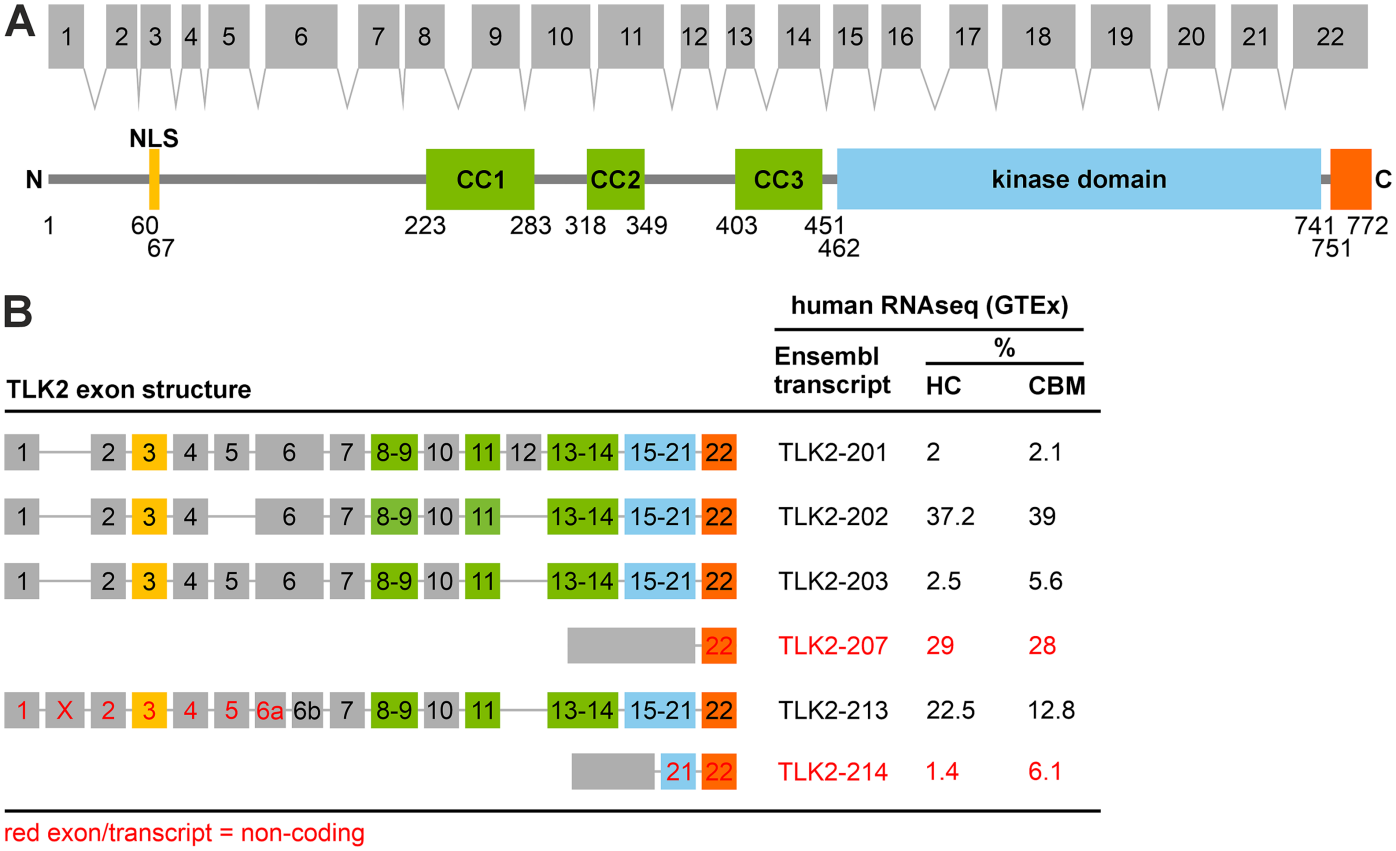
TLK2 has splice variant diversity in the human brain. **(A)** Exonic structure of human TLK2, mapped onto a schematic representation of its protein domains. NLS, nuclear localisation sequence; CC, coiled-coil domain. **(B)** Table of % TLK2 transcript abundance in the human hippocampus and cerebellum derived from GTEx RNAseq data. Exons and transcripts labeled in red are predicted to be non-protein coding. Only transcripts present >2% in either brain region are included.

Since the neuronal distribution and function of TLK2 has not been investigated, we first sought to characterise TLK2 transcript expression in the brain. Previous studies have observed tissue-specific transcripts of TLK2, in the mouse testis for example (Shalom and Don, 1999), and TLK2 immunoblots often have multiple bands that vary between tissues and cell lines (Kim et al., 2016b; Segura-Bayona et al., 2017). Such bands could result from post-translational modifications or alternative splicing of TLK2 or a combination. We began by interrogating publicly available human (GTEx) and mouse brain (Human Protein Atlas) transcriptomic data to characterise neuronal TLK2 transcript expression (Figures 1A,B), focussing on the hippocampus and cerebellum. We shall refer to human, mouse and rat TLK2 transcripts as hTLK2, mTLK2 and rTLK2, respectively. In the human brain, surprisingly the most abundant transcript was not canonical TLK2 (hTLK2-203), but a transcript lacking exon 5 (hTLK2-202; Figure 1B, Supplementary Figure S1). In the mouse brain, a full-length transcript corresponding to the canonical Ensembl TLK2 transcript was the most abundant transcript (mTLK2-204). We also identified further evolutionarily conserved splice variant diversity at the N-terminus of the human and mouse protein, encoded by exons 1–9. Not surprisingly, there were fewer alternative splicing events in exons encoding the middle coiled-coil domain and C-terminal kinase domain of TLK2, which are essential for dimerisation and catalytic activity, respectively, (Mortuza et al., 2018; Figure 1B; Supplementary Figure S2). Interestingly, splice variants lacking exon 3 in which the nuclear localisation sequence (NLS, Figure 1A) is located (Yamakawa et al., 1997) are also abundant (Figure 1B; Supplementary Figures S1, S2). This suggests that a pool of TLK2 is constitutively resident outside the nucleus. Henceforth in this study we shall refer to isoforms that include the NLS as ‘long TLK2’, and those lacking the NLS as ‘short TLK2’. In the human GTEx dataset it was observed that transcript hTLK2-207 represents nearly a third of TLK2 mRNA expression, but is predicted to be subject to nonsense mediated decay due to an intron retention at exon 21 (Figure 1B).

**Figure 2:**
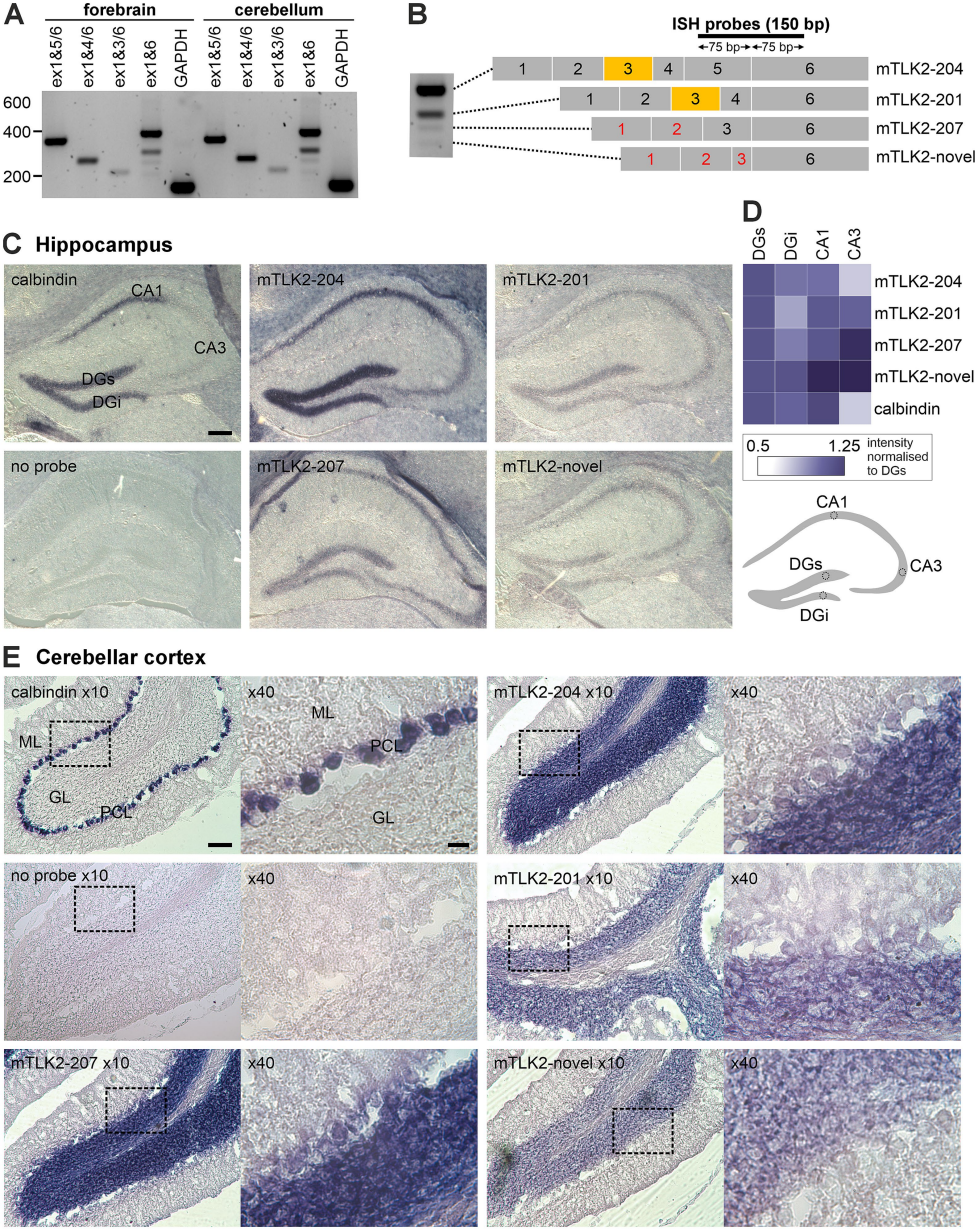
Multiple isoforms of TLK2 are expressed in mouse forebrain and cerebellum. **(A)** RT-PCR of TLK2 splice variants from mouse forebrain and cerebellum cDNA. Transcript specific primer pairs or pan exon 1–6 primers. **(B)** Left, lane from PCR of exon 1–6 primer pair. The four bands were excised and sequenced. Right, exon structure of sequenced mouse brain splice variants, and location of *in situ* hybridization probes. *In situ* hybridization was performed on 25 μm frozen coronal **(C)** or sagittal **(E)** P56 mouse brain sections. The indicated 150 bp exon junction spanning probes were used to examine calbindin or TLK2 transcript expression in the hippocampus **(C)** or cerebellar cortex **(E)**. Representative images are shown from *n* = 3 sections from two mouse brains. Cerebellar cortex images are shown at x10 and x40 magnification, indicated by the insert box. DG, dentate gyrus; ML, molecular layer; GL, granule layer; PCL, Purkinje cell layer. Scale bars: hippocampus 10 dentate gyrus; 0 μm, cerebellar cortex x10, 200 μm; x40, 20 μm. **(D)** Heatmap depicting quantification of TLK2 splice variant expression within the dentate gyrus, CA1 and CA3 sub-regions of the hippocampal formation. For each section, pixel intensity was measured in regions of interest (ROI), as indicated in the cartoon and normalized to the ROI located in the suprapyramidal blade of the dentate gyrus. Three sections were quantified for each probe.

To confirm the existence of alternative N-terminal TLK2 splice variants in the brain, we performed RT-PCR of mouse brain TLK2 mRNA. The annotated transcripts of mouse TLK2 have splice diversity within exons 1–6 (Figure 2A; Supplementary Figure S2). We first amplified and sequenced all N-terminal splice variants in mouse forebrain or cerebellar cDNA using transcript-specific primers and also a pair of ‘pan isoform’ primers located in exon 1 (forward) and exon 6 (reverse), which yielded four bands (Figure 2A). Three of the bands corresponded to the 5′ of annotated isoforms, two of which are predicted to contain the NLS (mTLK2-204 and -201) and one that lacks the NLS due to an alternative start site in exon 6 (mTLK2-207). We also sequenced a novel isoform that has a truncated exon 3, lacks exon 4 and 5 and would also not encode the NLS (mTLK2-novel; Figure 2B; Supplementary Figure S2). We next used *in situ* hybridisation to observe whether there is any cell type specificity in the expression of these TLK2 isoforms in the brain. All mouse TLK2 transcripts express exon 6 and combinations of exons 1–5, therefore we designed a suite of short 150 bp splice junction-spanning *in situ* probes anchored at the 3′ end by the first 75 bp complementary to exon 6 and containing a variable 5′ 75 bp complementary to exons 5, 4 or 3 (Figure 2B).

In coronal and sagittal mouse brain sections we examined the expression of TLK2 isoforms in the hippocampal formation and cerebellar cortex, respectively. A 150 bp exon junction spanning calbindin probe was used as a positive control, confirming calbindin expression in the Purkinje neurons of the cerebellar cortex and dentate gyrus and CA1 neurons of the hippocampus (Figures 2C,E). Low background staining was observed in the absence of an *in situ* probe (Figures 2C,E). In the coronal sections, hippocampal staining of all TLK2 isoform probes was detected in excitatory neurons of the dentate gyrus, CA1 and CA3 (Figure 2C). Although quantifying the intensity of staining between probes was not appropriate, using regions of interest placed in the DG, CA1 and CA3 fields, we quantified the relative intensity of staining of each TLK2 isoform in these hippocampal cell types and normalised the density to the signal in the suprapyramidal blade of the dentate gyrus (DGs; Figure 2C). The full length TLK2 (exons 1–6) probe, corresponding to the most abundant splice variant mTLK2-204, gave strong staining in the cell soma of the granule neurons in the DG and pyramidal neurons in CA1 and to a lesser extent in CA3 (Figures 2B, C). The other long TLK2 isoform (mTLK2-201) was less readily detected in the hippocampus, but quantification revealed differential expression in the two pyramidal blades of the DG, with the suprapyramidal blade having the highest intensity (Figure 2D). The relative staining of the shorter variant, mTLK2-207, was highest in CA1 neurons. In-line with the PCR (Figure 2A), staining of the novel short TLK2 isoform appeared weakest, with the highest intensity in CA1 and CA3 compared to the DG (Figures 2C, D).

In sagittal sections of the cerebellar cortex, staining of the TLK2 probes was readily detectable in the abundant granule neurons and to a lesser extent in Purkinje neurons (Figure 2E). The intensity of staining for the full length mTLK2-204 probe was similar to that of the shorter mTLK2-207 in the granule cell and Purkinje layers, whereas the mTLK2-201 and mTLK2-novel probes had weak granule layer staining and barely detectable staining of Purkinje neurons (Figure 2E). For all TLK2 probes, we failed to observe staining of inhibitory interneurons, resident in the molecular layer of the cerebellar cortex. It is likely the expression is beyond the detection level of our technique since a single-cell RNAseq study detected TLK2 transcripts in mouse cerebellar Golgi interneurons (Kozareva et al., 2021). Taken together, these data confirm the GTEx RNAseq data showing alternative splice variants of TLK2 are expressed in neurons of the mammalian brain.

### TLK2 is predominantly non-nuclear in adult mouse brain

**Figure 3:**
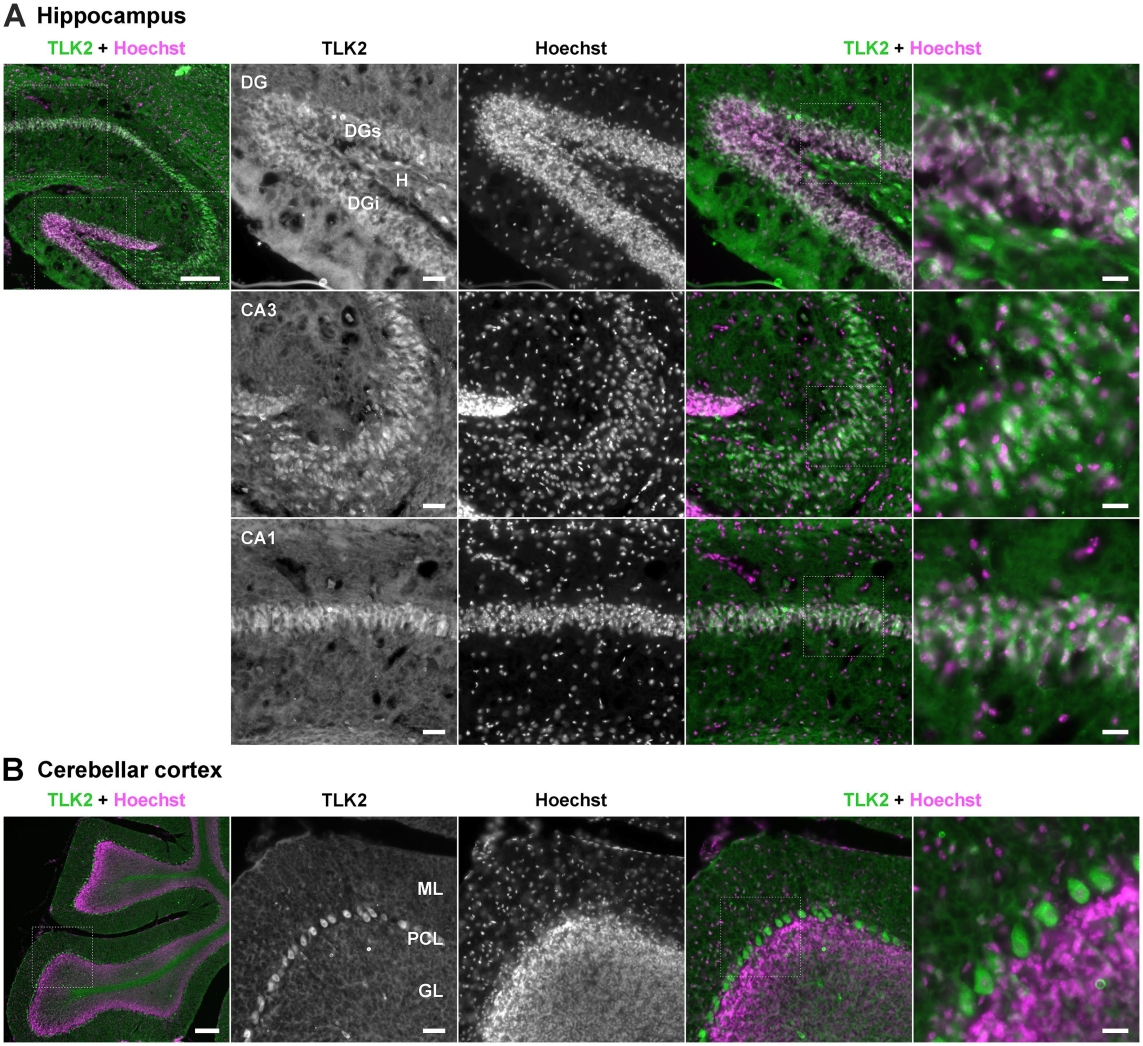
Neuronal TLK2 is predominantly non-nuclear in the adult mouse brain. Immunohistochemistry was performed on 25 μm frozen sagittal P56 mouse brain sections with anti-TLK2 (green) and Hoechst DNA stain (magenta). Representative images are shown from *n* = 3 sections from one mouse brain. Hippocampal **(A)** and cerebellar cortex **(B)** images were obtained with x5 (far left panel) and x20 (all other panels) objectives, with further magnified areas indicated by white dotted lines. The 20x images of the hippocampal formation focus on the dentate gyrus (DG); CA3 and CA1. H, hilus; ML, molecular layer; GL, granule layer; PCL, Purkinje cell layer. Scale bars: far left x5 composite panels, 200 μm; middle x20 panels, 50 μm; far right x20 magnified composite panels, 20 μm.

Having observed TLK2 transcript expression patterns in the mouse brain we used the same preparation to visualise the localisation of neuronal TLK2 protein. We performed immunohistochemistry with anti-TLK2 in the hippocampus and cerebellum of P56 frozen adult mouse sagittal sections (Figure 3). Secondary antibody controls revealed detectable background staining of the sections (Supplementary Figure S4) but there was clearly specific staining with anti-TLK2. A striking observation across the hippocampal and cerebellar staining was the weak TLK2 immunoreactivity that co-localised with Hoechst positive nuclei. Instead TLK2 appeared to be most localised in the cell cytoplasm, and often punctate (Figure 3). In the hippocampus, like the TLK2 *in situ* hybridisation, staining was most dense in the dentate gyrus, and cell layers of the CA1, 2, and 3 regions (Figure 3A). There was also diffuse staining in the stratum radiatum and other layers that are mainly composed of neuronal projections. In the dentate gyrus there was co-localisation of TLK2 with nuclei, but more intense staining within and between granule cell soma. Interestingly there were sparse larger cells of the hilus stained intensely with TLK2 in their cytoplasm, which was also true of cells in the CA3 and CA1 layers. In the cerebellar cortex, there was intense staining of TLK2 in the cell cytoplasm of Purkinje neurons, weak staining in their nuclei, and diffuse staining of their processes in the molecular layer (Figure 3B). Other sparse cells residing in the molecular layer, were also stained. The granule cell layer, which had stained strongly for TLK2 transcripts, had TLK2 immunoreactivity that appeared to be between the cell soma, perhaps representing granule cell processes or mossy fibre synapse glomeruli.

### Alternative splicing of TLK2 is regulated during neuronal differentiation

**Figure 4:**
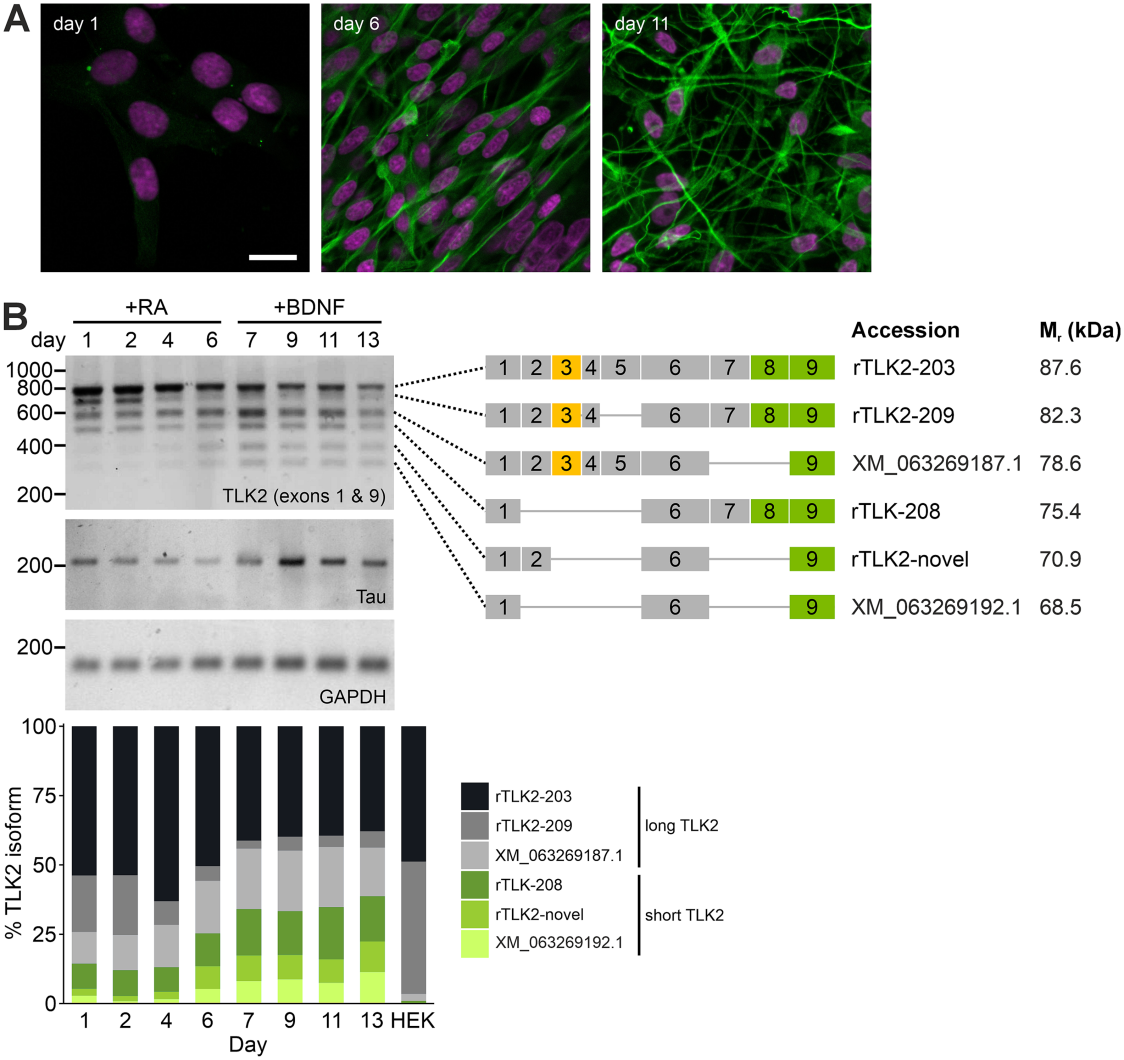
Alternative splicing of TLK2 is regulated during neuronal differentiation. B104 cultures were differentiated by sequential treatment with retinoic acid (RA) and brain derived neurotrophic factor (BDNF) as described in Materials and Methods. **(A)** Confocal images of B104 cultures at the indicated days of differentiation immunostained with β3-tubulin (green) and stained with DAPI (magenta). Scale bar = 20 μm. **(B)** cDNA was prepared from B104 cells at the indicated time points and subjected to PCR with primers for exons 1 and 9 of TLK2, Tau, and GAPDH. The six TLK2 bands obtained from the cells were excised and subjected to Sanger sequencing. The right-hand schematic summarises the exon structure of each sequence and any corresponding Ensembl or RefSeq transcripts that these align to and the predicted molecular mass of the encoded protein. The third heaviest PCR product yielded incomplete sequence and based on its mass is likely to represent RefSeq: XM_063269187.1. The bottom plot presents quantitative densitometry of each TLK2 RT-PCR product, expressed as a percentage of the total intensity at each timepoint.

**Figure 5:**
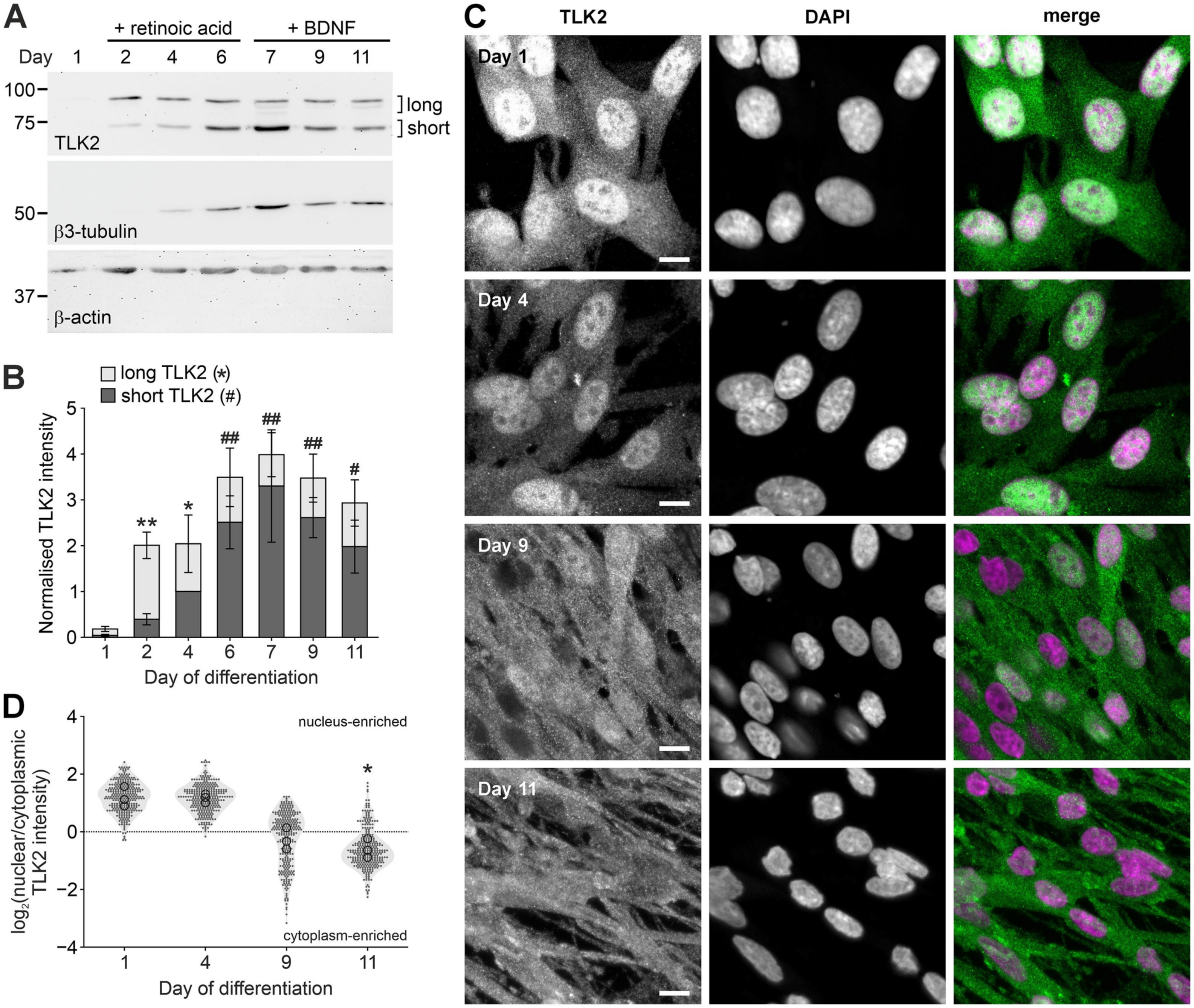
Neuronal differentiation alters the nuclear-cytoplasmic distribution of TLK2. B104 cells were lysed or fixed at the indicated timepoints during neuronal differentiation and subjected to immunoblotting with anti-TLK2, anti-β3-tubulin, or anti-actin **(A)** or immunocytochemistry with anti-TLK2 (1:1,000; green) and DAPI staining (purple). Scale bar = 10 μm **(C)**. **(B)** Quantification of TLK2 immunoblot band intensity from **(A)**, plotted as stacked bars, with long TLK2 in light grey and short TLK2 in dark grey. Data were normalized to the intensity of actin and then day 4 short TLK2 (*n* = 3 independent experiments). **(D)** TLK2 intensity was measured in regions of interest in the nucleus and cytoplasm of 100 cells from each timepoint (*n* = 3 independent experiments). Data are plotted as log_2_ (nuclear/cytoplasmic) TLK2 intensity at each timepoint (grey filled circles). Hollow black circles represent the mean of each replicate. For **(B,D)**, data were analyzed by Kruskal–Wallis two-tailed ANOVA and Dunn’s *post-hoc* test; *p < 0.05; **p < 0.01 compared to day 1. For significance in **(B)**, * = long TLK2 and # = short TLK2.

To date, our knowledge of TLK2 is mainly limited to its nuclear functions (Segura-Bayona and Stracker, 2019), although cell cycle-dependent shuttling of TLK2 between the nucleus and cytoplasm has been reported (Zhang et al., 1999). The observation of a non-nuclear pool of TLK2 in post-mitotic neurons has interesting implications for the role of TLK2 in the developing and mature brain. We hypothesised that cytoplasmic TLK2 in mature neurons arises from the upregulation of TLK2 isoforms lacking the NLS and/or the nuclear export of full length TLK2. To test this hypothesis, we adopted a neuronal differentiation model in the rat neuroblastoma cell line B104. Based on a previously published method we used the sequential application of retinoic acid (RA) and then BDNF with serum withdrawal over several days (Encinas et al., 2000), which produced robust neuronal morphology, expression of β3-tubulin protein (Figures 4A, 5A) and enhanced transcript expression of the neuronal marker tau (Figure 4B). Rat TLK2 transcript annotations in Ensembl and Genbank broadly map onto those found in human and mouse, with splice diversity predominantly within exons 1–9. To capture the diversity of 5′ rTLK2 splice variants during differentiation we performed RT-PCR on B104 cDNA samples from day 1–13 with primers located in exons 1 (forward) and 9 (reverse). This yielded six bands (Figure 4B), which were sequenced (Supplementary Figure S3). Figure 4B summarises the exon structure of each TLK2 PCR product, their Ensembl or RefSeq accession and the predicted mass of the protein they encode. The longest PCR product mapped onto rTLK2-203, the full-length canonical rat TLK2. The second product lacked exon 5 (rTLK2-209), representing a splicing event also observed in human and mouse transcripts. Sequencing the third band yielded partial coverage of exons 1–3 and then a mix that we were unable to deconvolve (Figure 4B; Supplementary Figure S3). Based on the mass of the PCR product, the most likely matching transcript lacks exons 7 and 8 (RefSeq: XM_063269187). The three smallest PCR products corresponded to TLK2 isoforms lacking exons 3, 4 and 5, with alternative splicing of exons 2, 7 and 8 (Figure 4B). The fifth band, containing exons 1, 2, 6, and 9 represents a novel transcript. All three of these shorter transcripts are predicted to be protein coding and lack the NLS.

We observed a notable regulation of TLK2 isoform expression during neuronal differentiation of the B104 cells (Figure 4B). In undifferentiated cells the TLK2 isoforms are dominated by the long transcripts (rTLK-203; rTLK-209; XM_063269187.1) and the longest of the short isoforms that lacks the NLS (rTLK2-208). After the addition of RA, the expression of the long rTLK-209 isoform is rapidly downregulated (Figure 4B). The subsequent addition of BDNF in serum-free media induced an upregulation of the shortest variants (TLK2-novel and XM_063269192.1) and a concomitant downregulation of the longer isoforms. At the end of the differentiation protocol, the TLK2 isoform profile was a broader mix of long and short variants with similar levels of expression (Figure 4B).

To establish the corresponding pattern of TLK2 isoform protein expression during differentiation, B104 whole cell lysates from the differentiation timecourse experiment were subjected to immunoblotting with a C-terminal reactive antibody, for which the epitope is predicted to be present in all major splice variants of TLK2 (Figure 5A). Based on the predicted molecular weight of the proteins encoded by the TLK2 transcripts identified in B104 cells (see Figure 4B), we expected that the long isoforms would migrate in the range 82–85 kDa, while the shorter isoforms lacking the NLS would migrate at 68–75 kDa. Indeed, two groups of bands were observed at approximately 85 kDa and 70 kDa, and their expression was regulated during B104 differentiation, broadly correlating with the expression of the long and short TLK2-transcripts observed in Figure 4. Quantification of replicate immunoblots by densitometry revealed that the 85 kDa band had a biphasic expression during differentiation, which reduced during RA treatment and then stabilised during BDNF treatment (Figures 5A,B). The ~70 kDa band increased in expression during differentiation, with this band becoming the dominant isoform in the fully differentiated cells (Figures 5A,B). Taken together these data suggest that neuronal differentiation enhances expression of one or more TLK2 isoforms that lack the NLS and is predicted to be cytoplasmic.

### Cytoplasmic localisation of TLK2 in differentiated neurons

Immunofluorescent staining of B104 cells at 1, 4, 9 and 11 days of differentiation with the TLK2 C-terminal antiserum, revealed a time-dependent relocalisation of TLK2 from the nucleus to the cytoplasm (Figure 5C). We quantified its subcellular localisation by measuring the intensity of TLK2 staining in regions of interest within the cytoplasm and nucleus of the cells. The plot of log_2_ (cytoplasm/nucleus) in Figure 5D revealed that TLK2 is significantly enriched in the cytoplasm at day 11 compared to day 1.

**Figure 6:**
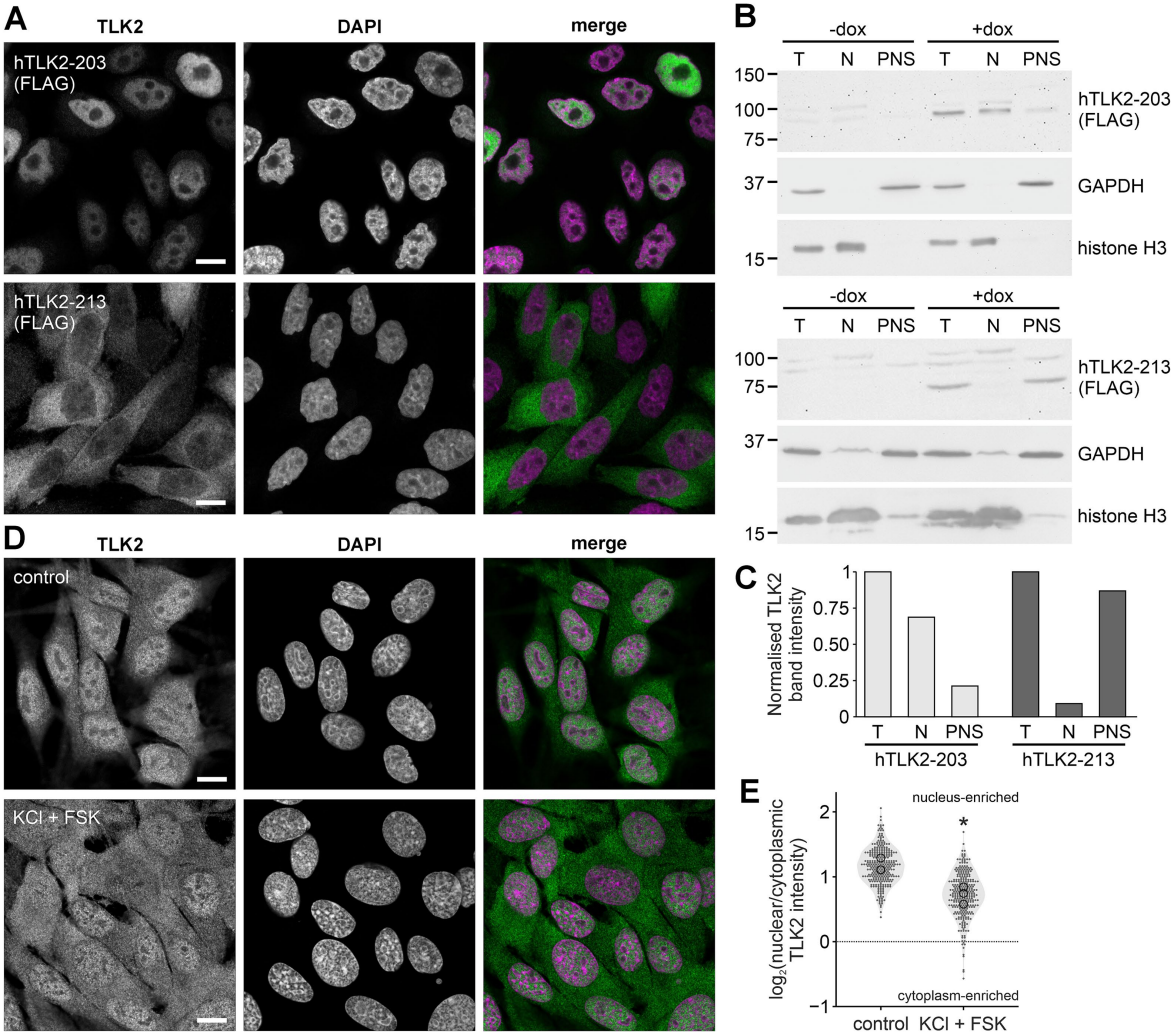
Activity-dependent redistribution of TLK2. Inducible Flp-in SK-N-SH cells expressing FLAG-tagged long hTLK2-203 or short hTLK2-213 were treated with or without 1 μg/mL doxycycline (DOX) for 48 h. **(A)** Representative immunofluorescence images of DOX-treated cells stained with anti-FLAG (1:1,000) and DAPI. Scale bar = 10 μm. **(B)** Cells plated in dishes were lysed (T) and subject to subcellular fractionation to yield a nuclear pellet (N) and postnuclear supernatant (PNS). The samples were subjected to immunoblotting with anti-FLAG, anti-GAPDH and anti-histone H3. **(C)** Quantification of FLAG-TLK2 band intensity from **(B)**. Bands in the nuclear and PNS fractions were normalised to the total levels (T) of hTLK2-203 or hTLK2-213. **(D)** B104 cells equilibrated in KREBS buffer were incubated for 1 h in KREBS buffer with or without (control) 100 mM KCl and 10 μM forskolin (FSK). Fixed cells were stained with anti-TLK2 and DAPI. Scale bar = 10 μm. **(E)** TLK2 intensity was measured in regions of interest in the nucleus and cytoplasm of 100 cells from each treatment (*n* = 3 experiments). Data are plotted as log_2_ (nuclear/cytoplasmic) TLK2 intensity at each timepoint (grey filled circles). Hollow black circles represent the mean of each replicate and were analysed by *t*-test; *p < 0.05 compared to control.

To confirm that TLK2 proteins encoded by splice variants lacking the NLS-containing exon 3 localise to the cytoplasm, we generated stable tet-inducible human neuroblastoma SK-N-SH T-REx-Flp-in cell lines that express either FLAG-TLK2 (180–750), corresponding to the predicted protein encoded by Ensembl isoform hTLK2-213 or full length FLAG-TLK2 corresponding to the protein product of the canonical Ensembl TLK2 full length isoform (hTLK2-203). Following a 48 h induction of TLK2 expression with doxycycline in these cell lines, we performed a crude subcellular fractionation (Figure 6B). The cells were subjected to hypotonic lysis and we isolated the pellet (enriched in nuclei) and post-nuclear supernatant. Immunoblotting with antibodies raised to compartment markers, histone H3 and GAPDH, confirmed enrichment of nuclear and cytoplasmic proteins, respectively, (Figure 6B). The immunostaining of anti-FLAG of the subcellular fractions and in fixed cells demonstrated that the N-terminal truncated kinase was exclusively localised to the cytoplasm, while the full length kinase is predominantly nuclear (Figures 5A–C). Taken together we propose that a cytoplasmic pool of TLK2 increases during neuronal differentiation due to two phenomena: an increase in the expression of a short TLK2 splice variant lacking the NLS and the export of full length TLK2 from the nucleus. This mechanism is consistent with our observation of a predominantly cytoplasmic localisation of TLK2 in mature neurons of the mouse brain.

### Nuclear-cytoplasmic shuttling of TLK2

In their description of the nuclear-cytoplasmic transport of TLK2, Zhang et al. (1999) discovered a yeast 2-hybrid interaction between a 14–3-3ζ bait and a C-terminal clone of TLK2 (amino acids 657–750). They found that 14–3-3ζ immunoprecipitated from cell lysates with TLK2 and a dominant negative 14–3-3 promoted the nuclear localisation of TLK2 (Zhang et al., 1999). Since 14–3-3 proteins act by binding a phosphorylated motif (Yaffe et al., 1997), we hypothesised that during neuronal differentiation, phosphorylation of TLK2 recruits 14–3-3, thus preventing nuclear import of TLK2 and restricting it to the cytoplasm. Using undifferentiated B104 cells, we attempted to mimic these signals to drive cytoplasmic localisation of endogenous TLK2 by stimulating kinase signalling with 100 mM KCl and 10 μM forskolin for 1 h. 100 mM KCl is known to depolarise neuroblastoma cell lines, mimicking synaptic activity, and should activate Ca^2+^-dependent intracellular signalling in addition to the MAP kinase pathway (Corrales et al., 2005). Forskolin, an adenylyl cyclase agonist, stimulates cAMP production and hence activates signalling downstream of PKA (cAMP-dependent protein kinase). Cells treated with KCl and forskolin together caused TLK2 to translocate from the nucleus to the cytoplasm as visualised by immunofluorescence (Figure 6D) and quantified by log_2_ (nucleus/cytoplasm) of TLK2 intensity (Figure 6E). As a positive control, under the same conditions we observed shuttling of TORC1/CRTC1 from the cytoplasm to the nucleus (Supplementary Figure S5), a process known to be dependent on Ca^2+^- and cAMP-dependent signalling (Ch’ng et al., 2012; Zhang et al., 2012). These data suggest the nucleocytoplasmic shuttling of TLK2 that occurs during neuronal differentiation might also be regulated in mature neurons by activity-dependent signalling.

## Discussion

We have undertaken the first characterisation of neuronal TLK2 expression and localisation. We found that TLK2 transcripts and protein are expressed in the hippocampus and cerebellum, brain regions that are affected in neurodevelopmental disorders linked with disrupted motor function and cognition. In addition to canonical full length TLK2, we observed splice diversity at the N-terminus of the protein, including variants that lack an NLS. The observation of TLK2 in the cytoplasm of mature mouse brain neurons was supported by a cell line model of neuronal differentiation in which cytoplasmic TLK2 increased during differentiation. This was driven by both enhanced expression of TLK2 variants lacking the NLS, and nuclear export of full length TLK2. We also found that the localisation of TLK2 can be regulated acutely by mimicking synaptic activity. Our data suggest future studies into the pathological mechanisms of MRD57 should consider cytoplasmic TLK2 signalling in developing and mature neurons.

### Expression and alternative splicing of neuronal TLK2

Our analysis of TLK2 transcripts in mouse brain, B104 cells, and publicly available RNAseq datasets revealed broad conservation of full length TLK2 and shorter transcripts lacking N-terminus-encoding exons. Interestingly the longest human Ensembl isoform, hTLK2-201, comprising 22 exons is weakly expressed, except in aorta and testis (Supplementary Figure S1), while the most abundant variant in brain and across the body is hTLK-202, which lacks exons 5 and 12. Indeed the other major isoforms that are expressed (hTLK-203 and hTLK-213) also lack exon 12. hTLK-213 is the major annotated short isoform that lacks the N-terminus and NLS and is the human homologue of the short mTLK2-207 isoform that was first identified in mouse testis (Shalom and Don, 1999). When we assessed the splice diversity of TLK2 transcripts in rat B104 cells during neuronal differentiation, we detected several isoforms that lack the NLS encoded by exon 3. When we immunoblotted TLK2 in B104 cells using an antibody with a C-terminal epitope, we observed bands that broadly correspond to long and short isoforms. However, due to the minor differences in molecular mass arising from the various splicing events it was not possible to correlate specific protein bands with specific splice variants.

Interestingly, human TLK1, which shares 78% homology with full length TLK2, also possesses a short isoform, TLK1B, lacking the N-terminus and hence the NLS. Constitutive expression of TLK1B is highest in testis, liver and lung, but low in brain (Li et al., 2001). Furthermore, TLK1B expression can be induced by genotoxic stress, such as ionising radiation, through translational regulation of an alternative start site (Li et al., 2001). Surprisingly, in contrast to short TLK2, TLK1B localises to the nucleus where it coordinates DNA repair, even in the absence of the TLK1 NLS (Li et al., 2001; Sunavala-Dossabhoy et al., 2005), suggesting TLK1B is targeted to the nucleus by an alternative mechanism.

Using splice junction-specific *in situ* probes, we were able to examine the relative distributions of long and short TLK2 transcripts in the mouse brain. TLK2 was expressed in the major cell types of the hippocampus and cerebellum. We observed differential expression of TLK2 isoforms in excitatory cell types of the hippocampal formation, with intriguing differences between CA1 and CA3 intensity (mTLK2-204) and the blades that comprise the dentate gyrus (mTLK2-201). It is not surprising that there are transcriptional differences between hippocampal cell types, which presumably define their function. Immunohistochemistry, also in sagittal mouse brain sections revealed TLK2 protein expression in the same regions and cell types as the *in situ* hybridisation. The TLK2 antibody intensely stained cerebellar Purkinje neurons and cells of the dentate gyrus, CA3 and CA1 layers in the hippocampus, with weaker diffuse staining in the molecular layers of both regions. In all cell types the TLK2 staining was more intense in the cytoplasm than the nucleus. Since the TLK2 antibody is raised to a C-terminal epitope, we were unable to distinguish long or short isoforms. However, regardless of its isoform expression, these data suggest that in post-mitotic neurons TLK2 has important non-nuclear functions.

### Neuronal differentiation- and activity-dependent nucleocytoplasmic shuttling of TLK2

The B104 cell model allowed us to observe the loss of nuclear TLK2 staining during neuronal differentiation. Since we did not observe a significant concomitant reduction in protein expression of longer TLK2 isoforms, we concluded that TLK2 was shuttling from the nucleus to the cytoplasm during differentiation. The upregulation of short TLK2 isoform expression we observed is also likely to contribute to the enhancement of cytoplasmic TLK2. This was validated by creating stable SK-N-SH cells cell lines expressing an N-terminally truncated hTLK2 variant, which was confined to the cytoplasm. These data agree with the non-nuclear localisation of a short TLK2 variant that was overexpressed in AD293 cells (Mortuza et al., 2018). A question remains as to whether the dynamic localisation of TLK2 during neuronal differentiation is a cause or effect of the differentiation process. This could be tested by examining the effect of short or long TLK2 knockdown or overexpression upon neuroblastoma cell differentiation.

Our observation of long TLK2 export from the nucleus during differentiation is consistent with previous data on the cytoplasmic and nuclear distribution of TLK2. Using a C-terminal TLK2 monoclonal antibody, Zhang et al. (1999) demonstrated cell cycle-dependent shuttling of TLK2 in cultures of synchronised 3 T3 cells. During G1 phase, TLK2 was predominantly outside the nucleus and co-localised with vimentin, an intermediate filament protein. With the onset of S-phase TLK2 was observed to localise to the nuclear periphery and then within the nucleus during late G2 (Zhang et al., 1999). Thus, the nuclear export and cytoplasmic accumulation of TLK2 during neuronal differentiation correlates with these cells exiting the cell cycle. Mechanistically, Zhang et al. (1999) attributed the nucleocytoplasmic shuttling of TLK2 to an interaction with 14-3-3 proteins, with binding preventing TLK2 nuclear import. They identified a putative 14-3-3 interaction motif in the C-terminus (amino acids 747–753). Serine phosphorylation of 14-3-3 motifs is required for binding, but the phosphorylation of that motif in TLK2 has not been characterised. Since we observed translocation of TLK2 in response to KCl-induced depolarisation and FSK, it is possible that phosphorylation of TLK2 by a Ca^2+^- and/or cAMP-dependent kinase facilitates 14-3-3 binding and hence retains it in the cytoplasm. There are examples in the literature of transcription factors that require neuronal activity-dependent Ca^2+^ and cAMP signalling for nucleocytoplasmic shuttling, such as the transcriptional activator we used as a positive control, TORC1/CRTC1 (Ch’ng et al., 2012; Zhang et al., 2012). The functions of non-nuclear TLK2 have not been investigated, although its colocalisation with vimentin might suggest regulation of the cytoskeleton (Zhang et al., 1999). This is in-line with the Tousled homologue in *Drosophila*, which has been shown to genetically interact with *rho1* (Gregory et al., 2007), a cytoskeletal regulator, and the *tlk* mutant has altered microtubule and actin filament density in developing follicle cells (Yeh et al., 2015). Other cytoplasmic TLK2 interactors are lacking. Previous TLK2 immunoprecipitation or proximal interactome studies have been conducted in non-neuronal proliferating cell lines, where it might be expected that TLK2 is predominantly nuclear, and hence these have tended to identify nuclear binding partners (Pavinato et al., 2022; Segura-Bayona et al., 2017).

### The implications of neuronal TLK2 splice variants for MRD57

Our study raises mechanistic questions about the pathology of the neurodevelopmental disorder MRD57, in which patients are haploinsufficient for TLK2, predominantly through *de novo* mutations (Lelieveld et al., 2016; Pavinato et al., 2022; Reijnders et al., 2018; Woods et al., 2022). The important role of TLK2 in cell cycle regulation and DNA repair has led to the hypothesis that the proliferation and differentiation of neural progenitors is affected in MRD57 patient brain development (Segura-Bayona and Stracker, 2019). MRD57 phenotypes are presumably due to processes involving TLK2 that are not compensated by TLK1, such as non-nuclear functions in post-mitotic neurons. Surprisingly, the TLK2 knockout mouse did not display any cell cycle defects, due to compensation by TLK1, except in the placenta where TLK1 expression is low (Segura-Bayona et al., 2017). However, the knockout mouse might not be a good model for MRD57 as there were no reported brain or behavioural anomalies in the TLK2 conditional heterozygous or homozygous mutant mice (Segura-Bayona et al., 2017).

It is notable that MRD57 patient missense mutations have not been identified in exons 2, 4–7 or 12. This suggests that these regions are either not necessary for TLK2 function, or their mutation is tolerated through compensation by alternative splice variants lacking these exons. Relevant to our study, one patient has been identified with a homozygous substitution mutation (K55E) adjacent to the NLS (Töpf et al., 2020), and it would be interesting to study the localisation and nuclear shuttling of this TLK2 mutant in neurons. Furthermore, future studies of neuronal TLK2 should focus on identifying its neuronal cytoplasmic substrates to advance our understanding of TLK2 in developing and mature neurons and the pathology of MRD57.

## Data Availability

The original contributions presented in the study are publicly available. The novel TLK2 isoform sequences have been deposited in Genbank: PX778942 (novel mouse TLK2 variant) and PX939618 (novel rat TLK2 variant). Publicly available datasets were analyzed in this study. Human TLK2 transcript expression data was obtained from the GTEx Portal, accession: phs000424.v8.p2. Mouse brain TLK2 transcript data was obtained from the Human Protein Atlas (https://www.proteinatlas.org).

## Footnotes

1
https://software.broadinstitute.org/morpheus
