# Supplementary material for: Neuronal differentiation and activity drive nucleocytoplasmic shuttling of the intellectual disability kinase TLK2
Source: Front Cell Neurosci. 2026 Apr 7;20:1699735. doi: 10.3389/fncel.2026.1699735 (PMC13095609; doi:10.3389/fncel.2026.1699735)

## Supplementary Materials

**Figure S1. Human tissue TLK2 transcript distribution.** Heatmap of human TLK2 transcript expression in the indicated tissues, expressed as percent abundance. RNAseq data derived from the GTEx Portal, accession number phs000424.v8.p2. Transcripts highlighted in blue are also included in the table in Fig 1B.

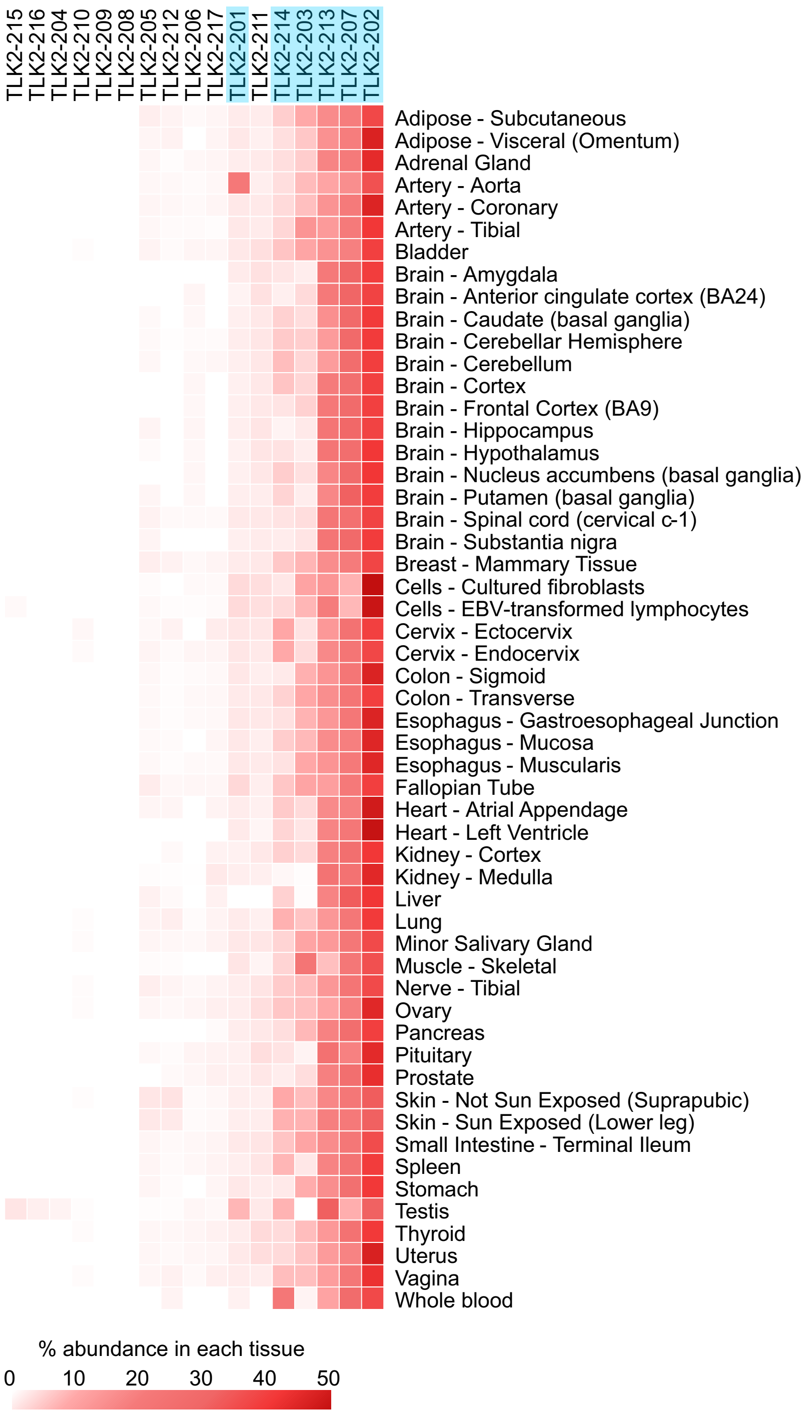

**Figure S2. TLK2 transcripts and their expression in the mouse brain. A**. Exonic structure of mouse TLK2, mapped onto a schematic representation of its protein domains. NLS, nuclear localisation sequence; CC, coiled-coil domain **B**. Table of % TLK2 transcript abundance in the mouse hippocampus and cerebellum derived from HPA RNAseq data plotted in C. Exons labelled in red are predicted to be non-protein coding. Only transcripts present >2% in either brain region are included. **C**. Heatmap of TLK2 transcript expression in the indicated brain regions, expressed as percent abundance. Data derived from HPA RNAseq dataset (Sjöstedt et al., 2020). Transcripts highlighted in blue are included in the table in **B**.

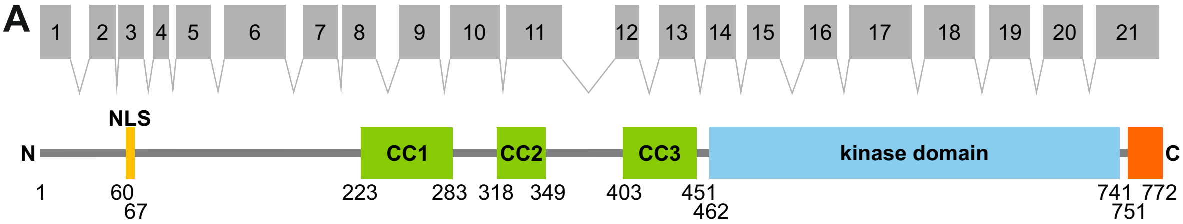


**B**

| TLK2 exon structure | Ensembl transcript | mouse RNAseq (HPA) % HC | mouse RNAseq (HPA) % CBM |
|---|---|---|---|
| 1 2 3 4 5 6 7 8-9 10 11 12-13 14-20 21 | TLK2-204 | 23.2 | 23.7 |
| 1 2 3 4 6 7 8-9 10 11 12-13 14-20 21 | TLK2-201 | 22.5 | 36.6 |
| 1 2 3a 3b 6 7 8-9 10 11 12-13 14-20 21 | TLK2-207 | 35 | 20.7 |
| 1 2 3 4 5 6 7 8-9 10 11 12-13 14-19 | TLK2-202 | 2.7 | 2.7 |
| 3 4 5 6 7 8 10 | TLK2-212 | 7.8 | 5.8 |
| 1 2 3 4 5 | TLK2-211 | 2.2 | 3.3 |
| 13 14-18 | TLK2-210 | 0.6 | 3 |

red exon/transcript = non-coding

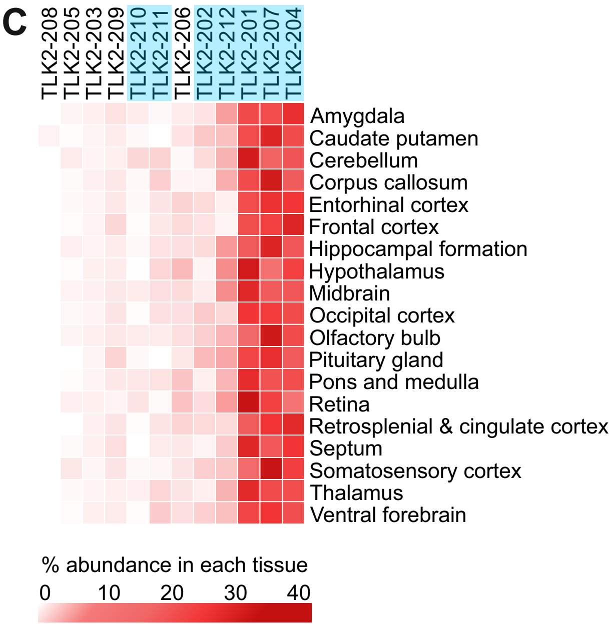

**Figure S3. (A) Sequencing of mouse TLK2 N-terminal splice variants.** cDNA prepared from mouse brain was subjected to PCR with primers located in exons 1 (mTLK2-exon1 sense) and 6 (mTLK2-exon6 anti) of mouse TLK2. The four PCR products obtained (see Figure 2) were excised from an agarose gel and subjected to Sanger sequencing. The sequences are presented as an alignment with each exon indicated by a different colour. The sequence of the novel variant was submitted to Genbank, accession number: PX778942.

```
                        Exon 1 (from 38 bp)
TLK2-204      GCAGGAATTACTAGAGGCCAGGTTCACTGGAGTTGGTGTAAGTAAGGGGCCACTCAACAG
TLK2-201      GCAGGAATTACTAGAGGCCAGGTTCACTGGAGTTGGTGTAAGTAAGGGGCCACTCAACAG
TLK2-207      GCAGGAATTACTAGAGGCCAGGTTCACTGGAGTTGGTGTAAGTAAGGGGCCACTCAGCAG
TLK2-novel    GCAGGAATTACTAGAGGCCAGGTTCACTGGAGTTGGTGTAAGTAAGGGACCACTCAACAG

                                 Exon 2
TLK2-204      TGAGTCTTCCAACCAGAGTCTGTGCAGCGTGGGGTCGTTGAGTGATAAAGAAGTAGAGAC
TLK2-201      TGAGTCTTCCAACCAGAGTCTGTGCAGCGTGGGGTCGTTGAGTGATAAAGAAGTAGAGAC
TLK2-207      TGAGTCTTCCAACCAGAGTCTGTGCAGCGTGGGGTCGTTGAGTGATAAAGAAGTAGAGAC
TLK2-novel    TGTGTCTTCCAACCAGAGTTTGTGCAGCGTGGGGTCCTTGAGTGATAAAGAAGTAGAGAC

                                 Exon 3
TLK2-204      TCCTGAGAAAAAGCAGAATGACCAGCGAAATCGGAAAAGGAAAGCCGAGCCATATGACAC
TLK2-201      TCCTGAGAAAAAGCAGAATGACCAGCGAAATCGGAAAAGGAAAGCCGAGCCATATGACAC
TLK2-207      TCCTGAGAAAAAGCAGAATGACCAGCGAAATCGGAAAAGGAAAGCCGAGCCATATGACAC
TLK2-novel    TCCTGAGAAAAAGCAGAATGACC-------------------------------------

                                 Exon 4
TLK2-204      TAGCCAAGGGAAAGGCACTCCTAGGGGACATAAAATTAGTGATTACTTTGAGTTTGCTGG
TLK2-201      TAGCCAAGGGAAAGGCACTCCTAGGGGACATAAAATTAGTGATTACTTTGAG--------
TLK2-207      TAGCCAAG----------------------------------------------------
TLK2-novel    ------------------------------------------------------------

                                 Exon 5
TLK2-204      GGGAAGCGGGCCAGGAACCAGCCCTGGCAGAAGTGTTCCACCAGTTGCACGATCCTCACC
TLK2-201      ------------------------------------------------------------
TLK2-207      ------------------------------------------------------------
TLK2-novel    ------------------------------------------------------------

                                               Exon 6
TLK2-204      GCAACATTCCTTATCCAATCCCTTACCGCGTCGAGCAGAACAGCCTCTGTATGGTTTAGA
TLK2-201      ----------------------------CGTCGAGCAGAACAGCCTCTGTATGGTTTAGA
TLK2-207      ----------------------------CGTCGAGCAGAACAGCCTCTGTATGGTTTAGA
TLK2-novel    ---------------------------GCTTCGAGTAGAACCGCCTCTGTATGGTTTAGA

TLK2-204      TGG---------
TLK2-201      TGGC--------
TLK2-207      TGGCAGTGCAGC
TLK2-novel    TGGCAGTGCAGC
```

**Figure S3. (B) Sequence alignment of rat B104 N-terminal TLK2 splice variants.** cDNA prepared from differentiating B104 cells was subjected to PCR with primers located in exons 1 (forward) and 9 (reverse) of TLK2. The six PCR products obtained (see Figure 4) were excised from an agarose gel and subjected to Sanger sequencing. The sequences obtained for bands 1,2,4 and 6 aligned with annotated TLK2 transcripts (rTLK2-203, rTLK2-209, rTLK2-208 and XM_063269192.1 respectively). Band 3 included exons 1, 2 and the start of exon 3 and then a mix of sequences, suggesting multiple transcripts of the same molecular mass. The novel sequence for band 5 was submitted to Genbank, accession number: PX939618. In the alignment below, each exon is indicated by a different colour.

```
                              Exon 1 (from 31 bp)
rTLK2-203      GGAGGCAGGAGTTACTGGAGGCCAGGTTCACTGGGGTTGGCGTAAGTAAGGGGCCACTCA
rTLK2-209      GGAGGCAGGAGTTACTGGAGGCCAGGTTCACTGGGGTTGGCGTAAGTAAGGGGCCACTCA
Band 3         -------------TCTGGAGG-CAGGTTCCTGGGGGTTGGCGTAAGTAAGGGGCCACTCA
rTLK2-208      GGAGGCAGGAGTTACTGGAGGCCAGGTTCACTGGGGTTGGCGTAAGTAAG----------
rTLK2-novel    GGAGGCAGGAGTTACTGGAGGCCAGGTTCACTGGGGTTGGCGTAAGTAAGGGGCCACTCA
XM_063269192.1 GGAGGCAGGAGTTACTGGAGGCCAGGTTCACTGGGGTTGGCGTAAGTAAG----------
                                   Exon 2
rTLK2-203      ACAGTGAGTCTTCCAACCAGAGTTTGTGCAGTGTGGGGTCCTTGAGTGATAAAGAAGTAG
rTLK2-209      ACAGTGAGTCTTCCAACCAGAGTTTGTGCAGTGTGGGGTCCTTGAGTGATAAAGAAGTAG
Band 3         ACAGTGAGTCTTCCAACCAGAGTTTGTGCAGTGTGGGGTCCTTGAGTGATAAAGAAGTAG
rTLK2-208      ------------------------------------------------------------
rTLK2-novel    ACAGTGAGTCTTCCAACCAGAGTTTGTGCAGTGTGGGGTCCTTGAGTGATAAAGAAGTAG
XM_063269192.1 ------------------------------------------------------------
                                   Exon 3
rTLK2-203      AGACTCCTGAGAAAAAGCAGAATGACCAGCGAAATCGGAAAAGAAAAGCTGAACCATATG
rTLK2-209      AGACTCCTGAGAAAAAGCAGAATGACCAGCGAAATCGGAAAAGAAAAGCTGAACCATATG
Band 3         AGACTCCAGA--------------------------------------------------
rTLK2-208      ------------------------------------------------------------
rTLK2-novel    AG----------------------------------------------------------
XM_063269192.1 ------------------------------------------------------------
                                   Exon 4
rTLK2-203      AAACTAGCCAAGGGAAAGGCACTCCTAGGGGACATAAAATTAGTGATTACTTTGAGTTTG
rTLK2-209      AAACTAGCCAAGGGAAAGGCACTCCTAGGGGACATAAAATTAGTGATTACTTTGAG----
Band 3         ------------------------------------------------------------
rTLK2-208      ------------------------------------------------------------
rTLK2-novel    ------------------------------------------------------------
XM_063269192.1 ------------------------------------------------------------
                                   Exon 5
rTLK2-203      CTGGGGGAAGCGGGCCAGGAACCAGCCCTGGCAGAAGTGTTCCACCAGTTGCACGATCCT
rTLK2-209      ------------------------------------------------------------
Band 3         ------------------------------------------------------------
rTLK2-208      ------------------------------------------------------------
rTLK2-novel    ------------------------------------------------------------
XM_063269192.1 ------------------------------------------------------------

rTLK2-203      CACCGCAACATTCCTTATCCAATCCCTTACCGCGTCGAGTAGAACAGCCTCTCTATGGTT
rTLK2-209      --------------------------------CGTCGAGTAGAACAGCCTCTCTATGGTT
Band 3         ------------------------------------------------------------
rTLK2-208      --------------------------------CGTCGAGTAGAACAGCCTCTCTATGGTT
rTLK2-novel    --------------------------------CGTCGAGTAGAACAGCCTCTCTATGGTT
XM_063269192.1 --------------------------------CGTCGAGTAGAACAGCCTCTCTATGGTT
```

```
                                     Exon 6
rTLK2-203      TAGATGGCAGTACTGCAAAGGAGGCCTCAGAAGAGCAGTCTGCCCTGCCAACCCTCATGT
rTLK2-209      TAGATGGCAGTACTGCAAAGGAGGCCTCAGAAGAGCAGTCTGCCCTGCCAACCCTCATGT
Band 3         ------------------------------------------------------------
rTLK2-208      TAGATGGCAGTACTGCAAAGGAGGCCTCAGAAGAGCAGTCTGCCCTGCCAACCCTCATGT
rTLK2-novel    TAGATGGCAGTACTGCAAAGGAGGCCTCAGAAGAGCAGTCTGCCCTGCCAACCCTCATGT
XM_063269192.1 TAGATGGCAGTACTGCAAAGGAGGCCTCAGAAGAGCAGTCTGCCCTGCCAACCCTCATGT

rTLK2-203      CAGTGATGTTAGCAAAACCTCGACTTGACACAGAGCAGTTAGCGCCAAGGGGAGCTGGCC
rTLK2-209      CAGTGATGTTAGCAAAACCTCGACTTGACACAGAGCAGTTAGCGCCAAGGGGAGCTGGCC
Band 3         ------------------------------------------------------------
rTLK2-208      CAGTGATGTTAGCAAAACCTCGACTTGACACAGAGCAGTTAGCGCCAAGGGGAGCTGGCC
rTLK2-novel    CAGTGATGTTAGCAAAACCTCGACTTGACACAGAGCAGTTAGCGCCAAGGGGAGCTGGCC
XM_063269192.1 CAGTGATGTTAGCAAAACCTCGACTTGACACAGAGCAGTTAGCGCCAAGGGGAGCTGGCC

rTLK2-203      TCTGCTTCACTTTCGTCTCTGCTCAACAAAACAGCCCTTCGTCCACGGGGTCTGGCAATA
rTLK2-209      TCTGCTTCACTTTCGTCTCTGCTCAACAAAACAGCCCTTCGTCCACGGGGTCTGGCAATA
Band 3         ------------------------------------------------------------
rTLK2-208      TCTGCTTCACTTTCGTCTCTGCTCAACAAAACAGCCCTTCGTCCACGGGGTCTGGCAATA
rTLK2-novel    TCTGCTTCACTTTCGTCTCT----------------------------------------
XM_063269192.1 TCTGCTTCACTTTCGTCTCT----------------------------------------
                                     Exon 7
rTLK2-203      CAGAACATTCTTGCAGCTCCCAGAAACAGATCTCCATCCAGCACAGGCAGACCCAGTCTG
rTLK2-209      CAGAACATTCTTGCAGCTCCCAGAAACAGATCTCCATCCAGCACAGGCAGACCCAGTCTG
Band 3         ------------------------------------------------------------
rTLK2-208      CAGAACATTCTTGCAGCTCCCAGAAACAGATCTCCATCCAGCACAGGCAGACCCAGTCTG
rTLK2-novel    ------------------------------------------------------------
XM_063269192.1 ------------------------------------------------------------
                                     Exon 8
rTLK2-203      ACCTCACAATAGAAAAAATATCTGCACTAGAAAACAGTAAGAACTCTGACTTAGAGAAGA
rTLK2-209      ACCTCACAATAGAAAAAATATCTGCACTAGAAAACAGTAAGAACTCTGACTTAGAGAAGA
Band 3         ------------------------------------------------------------
rTLK2-208      ACCTCACAATAGAAAAAATATCTGCACTAGAAAACAGTAAGAACTCTGACTTAGAGAAGA
rTLK2-novel    ------------------------------------------------------------
XM_063269192.1 ------------------------------------------------------------
                                                      Exon 9
rTLK2-203      AGGAAGGAAGAATAGATGATTTATTAAGAGCCAACTGTGATTTGAGACGACAGATAGATG
rTLK2-209      AGGAAGGAAGAATAGATGATTTATTAAGAGCCAACTGTGATTTGAGACGACAGATAGATG
Band 3         ------------------------------------------------------------
rTLK2-208      AGGAAGGAAGAATAGATGATTTATTAAGAGCCAACTGTGATTTGAGACGACAGATAGATG
rTLK2-novel    -----------------------------GCCAACTGTGATTTGAGACGACAGATAGATG
XM_063269192.1 -----------------------------GCCAACTGTGATTTGAGACGACAGATAGATG

rTLK2-203      AACAGCAAAAGATGCTAGAGA
rTLK2-209      AACAGCAAAAGATGCTAGAGA
Band 3         ---------------------
rTLK2-208      AACAGCAAAAGATGCTAGAGA
rTLK2-novel    AACAGCAAAAGATGCTAGAGA
XM_063269192.1 AACAGCAAAAGATGCTAGAGA
```

**Figure S4. Anti-rabbit 594 secondary controls for saggital mouse brain sections in Figure 3.** Immunohistochemistry was performed on 25 μm frozen sagittal P56 mouse brain sections with anti-goat anti-rabbit Alexa 594 and Hoechst DNA stain (magenta). Representative images are shown from n=3 sections from one mouse brain. Hippocampal (A) and cerebellar cortex (B) images were obtained with x5 (left panels) and x20 (right panels) objectives. Brightness and contrast settings in these images are the same as those used for the anti-TLK2 staining in Figure 3. Scale bars: x5 panels, 200 μm, x20 panels, 50 μm.

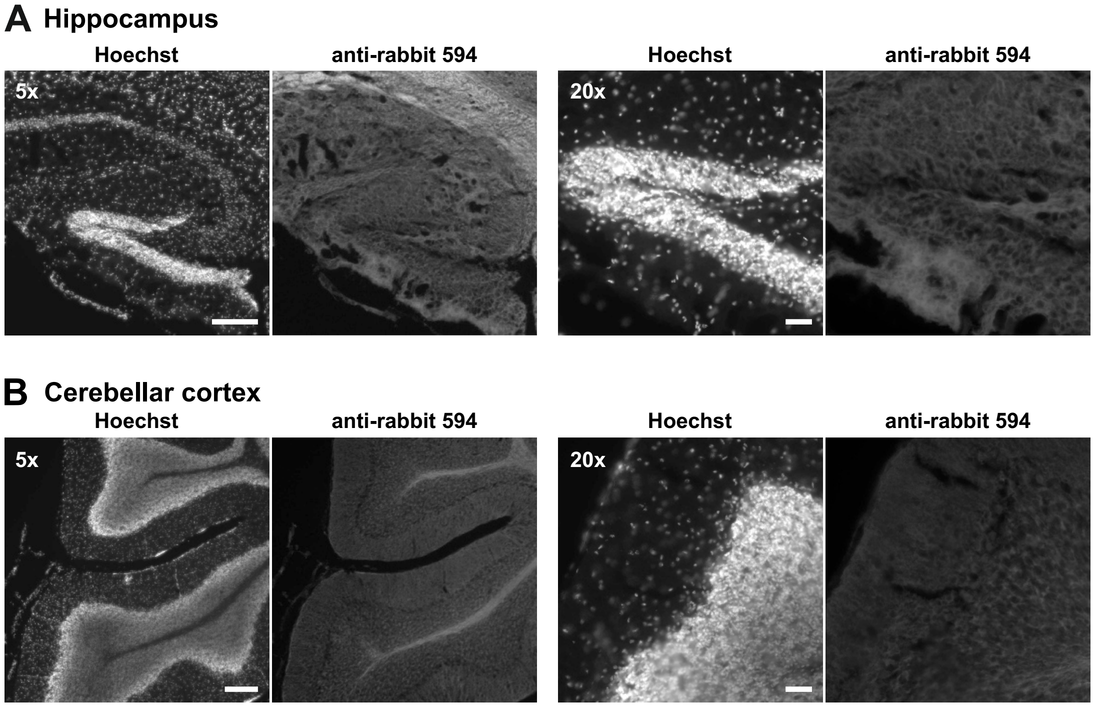

**Figure S5. Control for Figure 6D&E: TORC1/CRTC1 undergoes nucleocytoplasmic shuttling in response to KCl + forskolin treatment.** (A) B104 cells equilibrated in KREBS buffer were incubated for 1 h in KREBS buffer with or without (control) 100 mM KCl and 10 µM forskolin (FSK). Fixed cells were stained with anti-TORC1 and DAPI. Scale bar = 10 µm. (**D**) TORC1 intensity was measured in regions of interest in the nucleus and cytoplasm of 100 cells from each treatment (n=1 biological replicate, two coverslips/condition). Data are plotted as log2(nuclear/cytoplasmic) TORC1 intensity at each timepoint (grey filled circles). Hollow black circles represent the mean and were analysed by t-test (n=100); *** $p < 0.001$ compared to control.

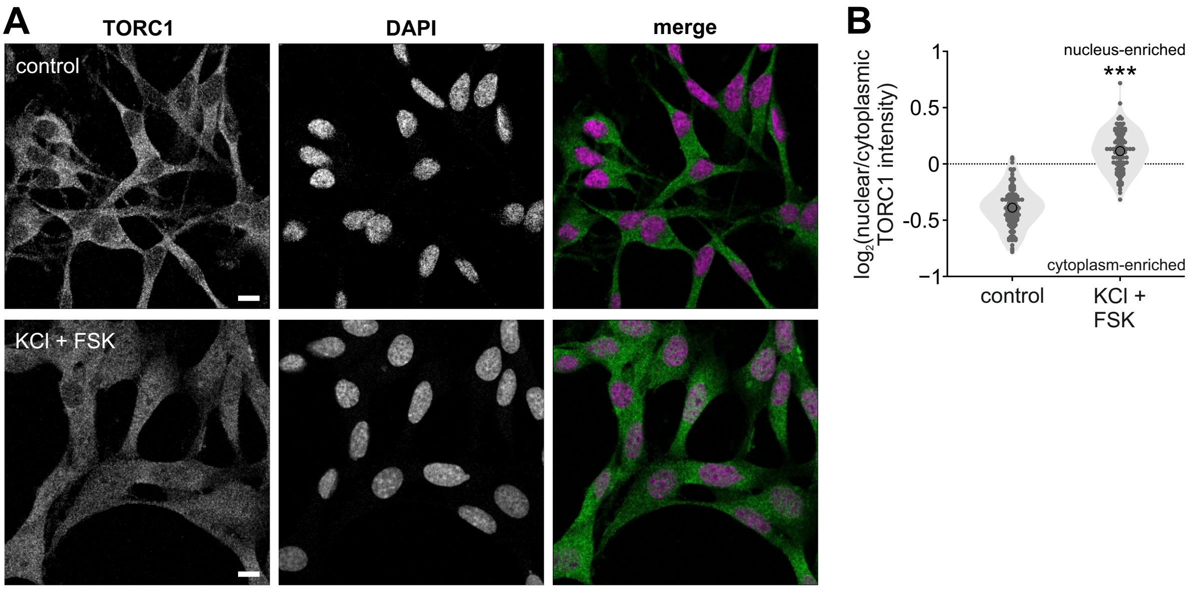

**Figure S6. Raw images of gels presented in figures.**

## Raw images from Figure 2

Gel-doc DNA image with crops for 2A and 2B

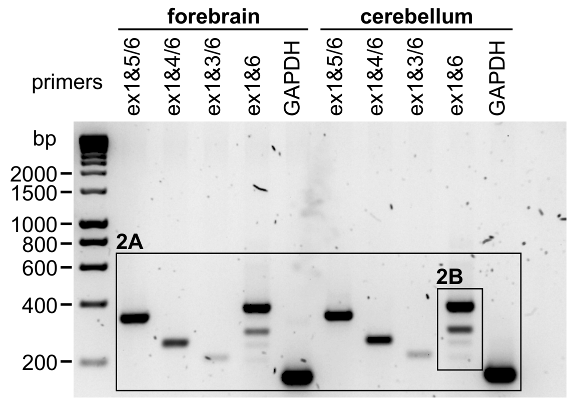


## Raw images from Figure 4A

Gel-doc DNA image with crops

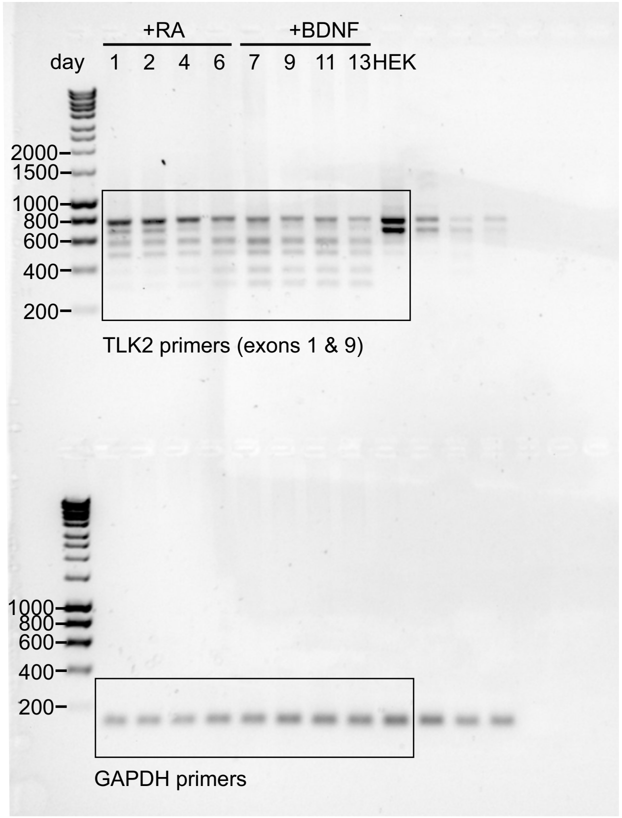


Gel-doc DNA image with crop

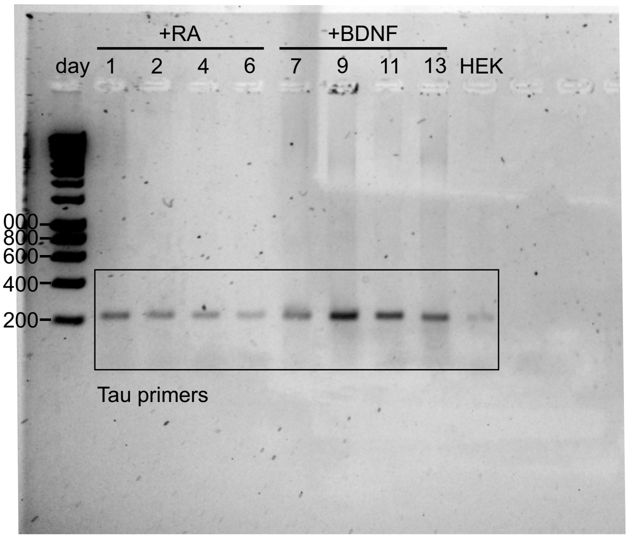

**Raw images from Figure 5A**

anti-TLK2 - membrane image

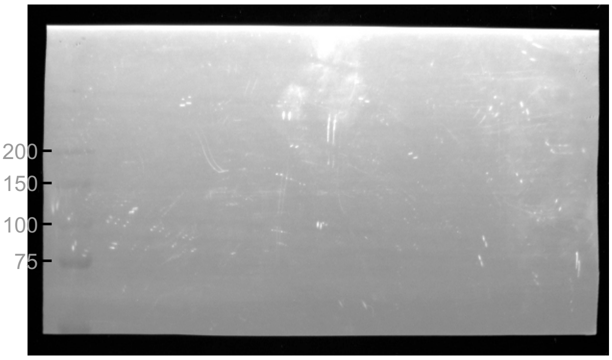


anti-TLK2 - iBright image with crop

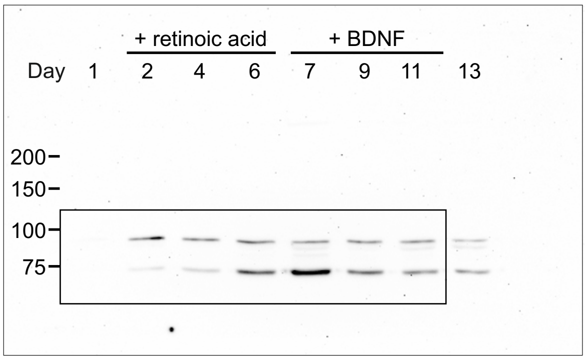


anti-actin - membrane image

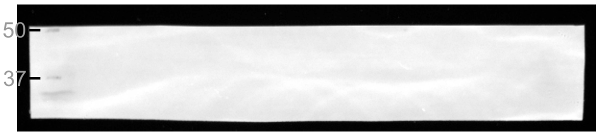


anti-actin - iBright image with crop

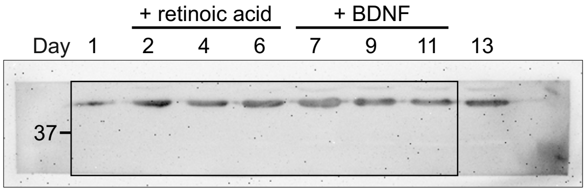


anti-β3-tubulin - membrane image

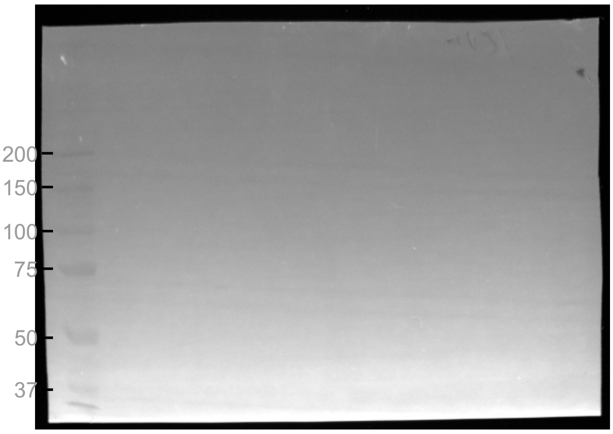


anti-β3-tubulin - iBright image with crop

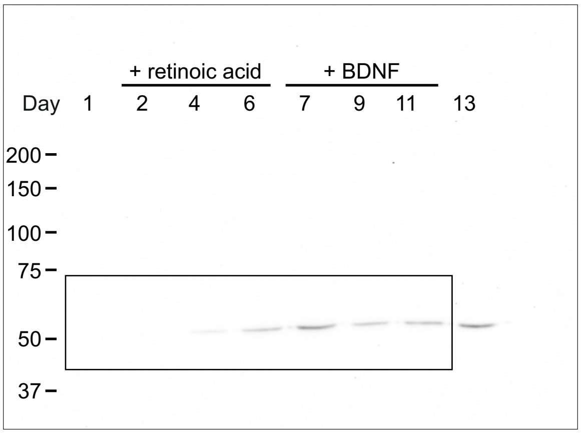

**Raw images from Figure 6B - top blots (TLK2-202)**

anti-FLAG - membrane image

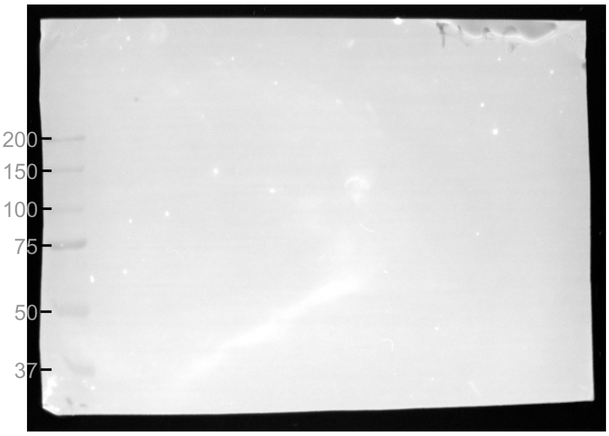


anti-FLAG - iBright image with crop

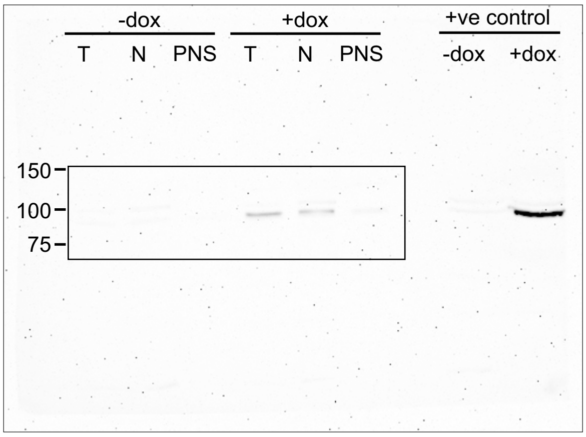


anti-GAPDH - membrane image

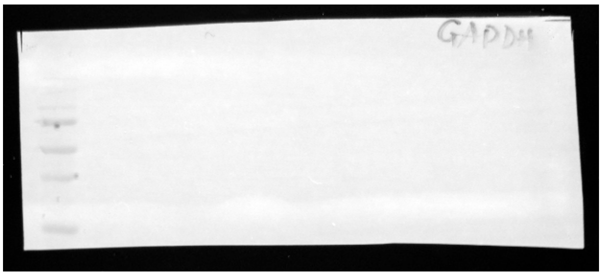


anti-GAPDH - iBright image with crop

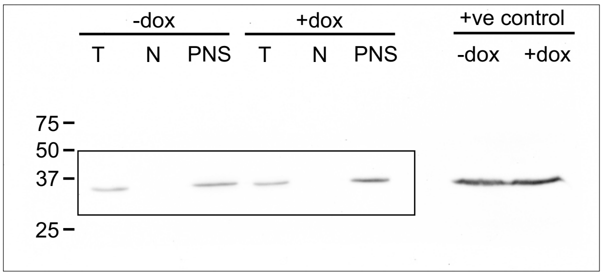


anti-histone H3 - membrane image

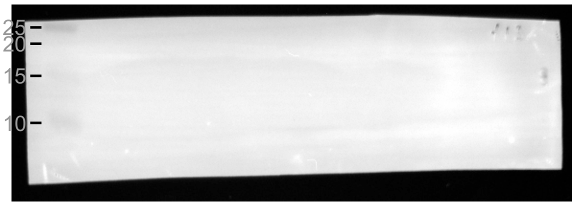


anti-histone H3 - iBright image with crop

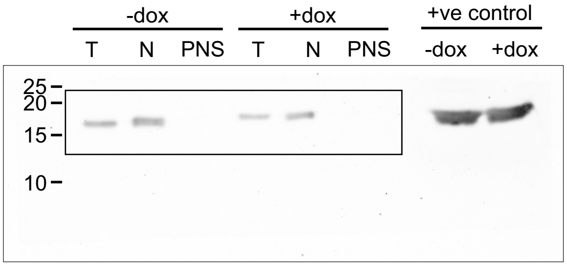

**Raw images from Figure 6B - bottom blots (TLK2-213)**

anti-FLAG - membrane image

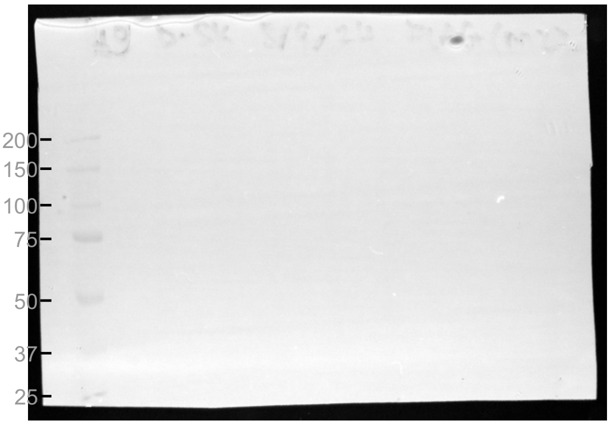


anti-FLAG - iBright image with crop

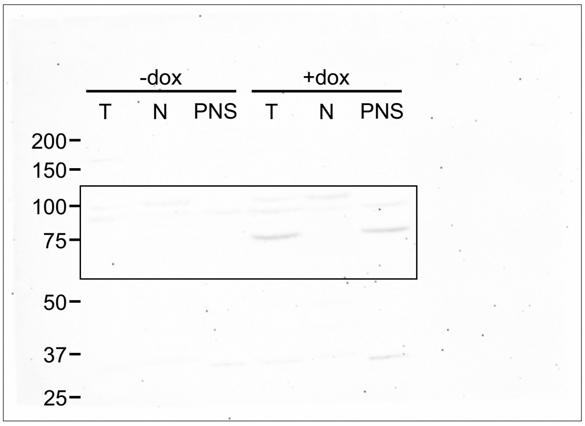


anti-GAPDH - membrane image

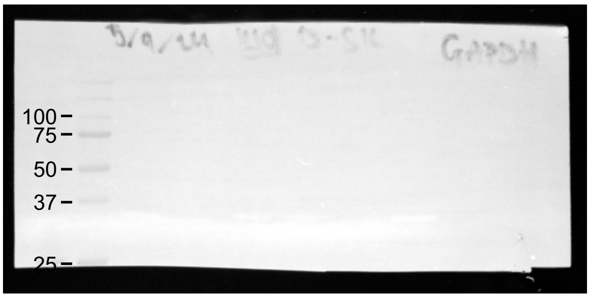


anti-GAPDH - iBright image with crop

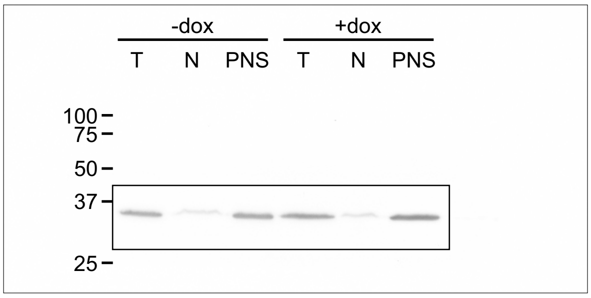


anti-histone H3 - membrane image

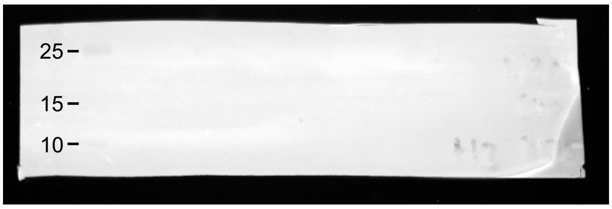


anti-histone H3 - iBright image with crop

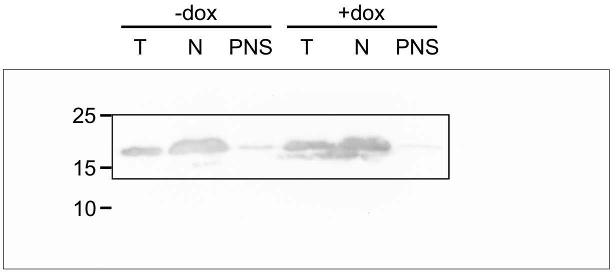

Supplement: Supplementary file 1 [file Data_Sheet_1.PDF]
